# Comparison of the diagnostic value of various microRNAs in blood for colorectal cancer: a systematic review and network meta-analysis

**DOI:** 10.1186/s12885-024-12528-8

**Published:** 2024-06-26

**Authors:** Jianhao Xu, Lanfen Pan, Dan Wu, Liqian Yao, Wenqian Jiang, Jiarui Min, Song Xu, Zhiyong Deng

**Affiliations:** 1 Department of Pathology, Kunshan First People’s Hospital Affiliated to Jiangsu University, Kunshan, Jiangsu China; 2 Immunopathology Innovation Team, Kunshan First People’s Hospital Affiliated to Jiangsu University, Kunshan, Jiangsu China

**Keywords:** Blood microRNAs, Tumor biomarkers, Colorectal cancer, Network meta-analysis

## Abstract

**Background:**

Despite the existence of numerous studies investigating the diagnostic potential of blood microRNAs for colorectal cancer, the microRNAs under consideration vary widely, and comparative analysis of their diagnostic value is lacking. Consequently, this systematic review aims to identify the most effective microRNA blood tumor markers to enhance clinical decision-making in colorectal cancer screening.

**Method:**

A comprehensive search of databases, including PubMed, Embase, Web of Science, Scopus, and Cochrane, was conducted to identify case‒control or cohort studies that examined the diagnostic value of peripheral blood microRNAs in colorectal cancer. Studies were included if they provided sensitivity and specificity data, were published in English and were available between January 1, 2000, and February 10, 2023. The Critical Appraisal Skills Programme (CASP) checklist was employed for quality assessment. A Bayesian network meta-analysis was performed to estimate combined risk ratios (RRs) and 95% confidence intervals (CIs), with results presented via rankograms. This study is registered with the International Platform of Registered Systematic Review and Meta-analysis Protocols (INPLASY), 202,380,092.

**Results:**

From an initial pool of 2254 records, 79 met the inclusion criteria, encompassing a total of 90 microRNAs. The seven most frequently studied microRNAs (43 records) were selected for inclusion, all of which demonstrated moderate to high quality. miR-23, miR-92, and miR-21 exhibited the highest sensitivity and accuracy, outperforming traditional tumor markers CA19-9 and CEA in terms of RR values and 95% CI for both sensitivity and accuracy. With the exception of miR-17, no significant difference was observed between each microRNA and CA19-9 and CEA in terms of specificity.

**Conclusions:**

Among the most extensively researched blood microRNAs, miR-23, miR-92, and miR-21 demonstrated superior diagnostic value for colorectal cancer due to their exceptional sensitivity and accuracy. This systematic review and network meta-analysis may serve as a valuable reference for the clinical selection of microRNAs as tumor biomarkers.

**Supplementary Information:**

The online version contains supplementary material available at 10.1186/s12885-024-12528-8.

## Background

Colorectal cancer, which ranks third in global cancer prevalence and second in mortality, presents a significant health challenge [1]. While developed regions such as Europe and the United States have observed a decline in incidence and mortality rates due to accessible screening and early treatment, the opposite trend is evident in certain low- and middle-income countries, where diagnosis and treatment are lacking [2]. In addition, the multi-drug resistance of cancer also makes the treatment increasingly difficult [3]. Thus, early detection and treatment are pivotal in enhancing colorectal cancer survival rates.

Peripheral blood tumor marker testing, a noninvasive procedure requiring only a patient’s peripheral blood sample, offers a viable alternative to tissue biopsy and imaging. This method is not only easy and quick to administer but also eliminates the need for preoperative preparation and recovery time, making it highly generalizable.

MicroRNA, as a peripheral blood tumor marker, holds several competitive advantages over other tumor markers. First, microRNAs are highly specific, with distinct miRNA expression patterns associated with different cancer types [4]. Second, they are stable, facilitating easy collection, storage, and transportation without degradation [5]. Third, microRNAs are highly sensitive, potentially offering more accurate early tumor detection than traditional tumor markers [6]. Finally, microRNA detection is versatile and useful not only for early cancer diagnosis but also for predicting treatment effects and prognosis assessment [7]. Consequently, the potential of miRNAs as novel biomarkers in early colorectal cancer diagnosis has been extensively researched, providing a scientific foundation for their clinical application.

However, the practical application of miRNAs necessitates the identification of the most diagnostically valuable miRNAs by clinicians, given the wide variety of miRNAs studied. The lack of direct comparison among various miRNAs and the inability of existing systematic reviews to determine the most diagnostically valuable miRNA necessitates an indirect comparison of the diagnostic value of different miRNAs through Bayesian network meta-analysis.

This study aims to identify the most diagnostically valuable microRNAs as blood tumor markers for colorectal cancer detection through network meta-analysis.

## Methods

The systematic reviews of observational studies were executed by the PRISMA guidelines (Preferred Reporting Items for Systematic Reviews and Meta-analysis) [8], with the study protocol registered under INPLASY202380092 [9].

### Eligibility criteria and PICO definition

Participants: The diagnostic test population was bifurcated into two groups, namely, patients diagnosed with colorectal cancer and healthy individuals.

Intervention: Pretreatment levels of microRNA in the peripheral blood of patients.

Comparison: Clinical pathological results as the gold standard test.

Study Design: Cohort or case‒control studies.

Outcome: Sensitivity and specificity.

Exclusion Criteria: Studies were excluded based on the following criteria: (1) To reflect current clinical practice, the timeliness of research is included in the exclusion criteria. Studies published before 2000 will be excluded [10, 11]. (2) Studies that were not published in the English language. (3) Manuscripts and conference abstracts that remained unpublished. (4) Studies that failed to report on either sensitivity or specificity.

### Information sources

On April 14, 2024, a subsequent ‘snowball’ search was conducted. This involved scrutinizing the reference lists of publications that were eligible for a full-text review and utilizing Google Scholar to discover and scrutinize studies that cited them, with the aim of identifying further studies.

### Search strategy

The following key words and MeSH terms (medical subject heading) in PubMed were used to find the related articles:

(1)Search: (“Colorectal Neoplasms“[Mesh]) OR (Colorectal cancer).

(2)Search: (“Biomarkers/blood“[Mesh]) OR ((“MicroRNAs“[Mesh]) OR (((((((((((((((((MicroRNA) OR (miRNAs)) OR (MicroRNA)) OR (RNA, Micro)) OR (miRNA)) OR (Primary MicroRNA)) OR (MicroRNA, Primary)) OR (Primary miRNA)) OR (miRNA, Primary)) OR (pri-miRNA)) OR (pri miRNA)) OR (RNA, Small Temporal)) OR (Temporal RNA, Small)) OR (stRNA)) OR (Small Temporal RNA)) OR (pre-miRNA)) OR (pre miRNA)))

(3)Search: (“Early Detection of Cancer“[Mesh]) OR (“Sensitivity and Specificity“[Mesh])

Search #1 AND #2 AND #3.

### Selection process

The selection process involved a rigorous review of titles and abstracts by three independent researchers (XJH, PLF, WD). Discrepancies were resolved through discussion until consensus was reached. The researchers then worked in pairs to independently screen the titles and abstracts of all retrieved articles. In cases of disagreement, a third researcher was consulted to make the final decision. The researchers also selected 7 microRNAs with a high number of cases for the net meta-analysis from a total of 106 microRNAs obtained by the nadir criteria screening.

### Data collection process and data items

Two review authors (XS and DZY) independently extracted data from eligible studies using a custom-designed data extraction table. The extracted data were compared, and any inconsistencies were resolved through discussion. When any of the above information was unclear, we contacted the report authors to provide further details.

Eligible results included sensitivity, specificity, accuracy, ROC (receiver operating characteristics) curve and area under the curve for peripheral blood microRNA diagnosis. For some multi-arm studies that do not directly provide the sensitivity and specificity of a miRNA, the decision threshold of the prediction model is achieved by the commonly used method of “maximizing the Jorden index“ [12]. Measurements were taken at the time point for all patients whose samples were collected before any treatment.

### Study risk of bias assessment

The potential bias in the studies was evaluated using a scoring system grounded on the Critical Appraisal Skills Programme (CASP) checklist, designed explicitly for diagnostic studies [13]. The CASP checklist has 12 questions to help understand a cohort or case-control study, which was independently applied to each of the included studies by the two review authors (XJH and PLF), who documented supporting information and the reasoning behind their bias risk judgment for each domain (low; high; some concerns). Any discrepancies in bias risk judgments or the reasoning behind these judgments were resolved through discussion until a consensus was reached between the two review authors. If necessary, a third review author acted as an arbitrator.

### Effect measures and synthesis methods

This review assesses the diagnostic value of microRNAs across three dimensions: sensitivity, specificity, and accuracy. Studies that satisfied the inclusion and exclusion criteria and provided data for all three dimensions were deemed suitable for synthesis. The primary steps of the analysis in this paper are as follows: Firstly, sensitivity, specificity, and accuracy were compared with the estimated odds ratios (ORs) and 95% confidence intervals (CI) using a random-effects model. Also, I-square tests were run to detect the amount of heterogeneity for each pairwise meta-analysis. Secondly, a network graph was constructed to elucidate the interconnections among the microRNAs being scrutinized. This graphical representation was instrumental in enabling comparisons, both direct and indirect. The scale of nodes and edges within the network was determined by the sample size and the number of studies contributing to each comparison. The Bayesian network meta-analysis was applied to estimate the combined effect sizes of these comparisons, harnessing Markov chain Monte Carlo (MCMC) simulations. Noninformative priors underpinned these simulations to estimate the magnitude and precision of effects. The convergence of the model was confirmed after executing four separate chains and a preliminary burn-in phase consisting of 10,000 simulations. The probability distributions were derived from a subsequent series of 50,000 simulations [14]. The RRs and 95% CIs were calculated to articulate our results, interpreting intervals that did not encompass the value of one as statistically significant. The heterogeneity was assessed using the I^2^ statistic, calculated from the MCMC samples, considering values above 50% indicative of considerable heterogeneity among the cohorts compared [15]. The relative efficacy of each microRNA was assessed through nanograms, which depicted the cumulative probability of each microRNA’s effectiveness, ranging from the most to the least effective. The node-splitting methods were further employed to test the network meta-analysis’s underlying assumption of consistency between direct and indirect evidence [16].

All computations were executed using R-4.0.3, with the “gemtc” and “netmeta” packages for network meta-analysis and the “mada” package for traditional meta-analysis. Furthermore, the mvmeta package was employed to plot inconsistency analyses and publication bias.

## Results

### Selection and characteristics of the study

**Fig. 1:**
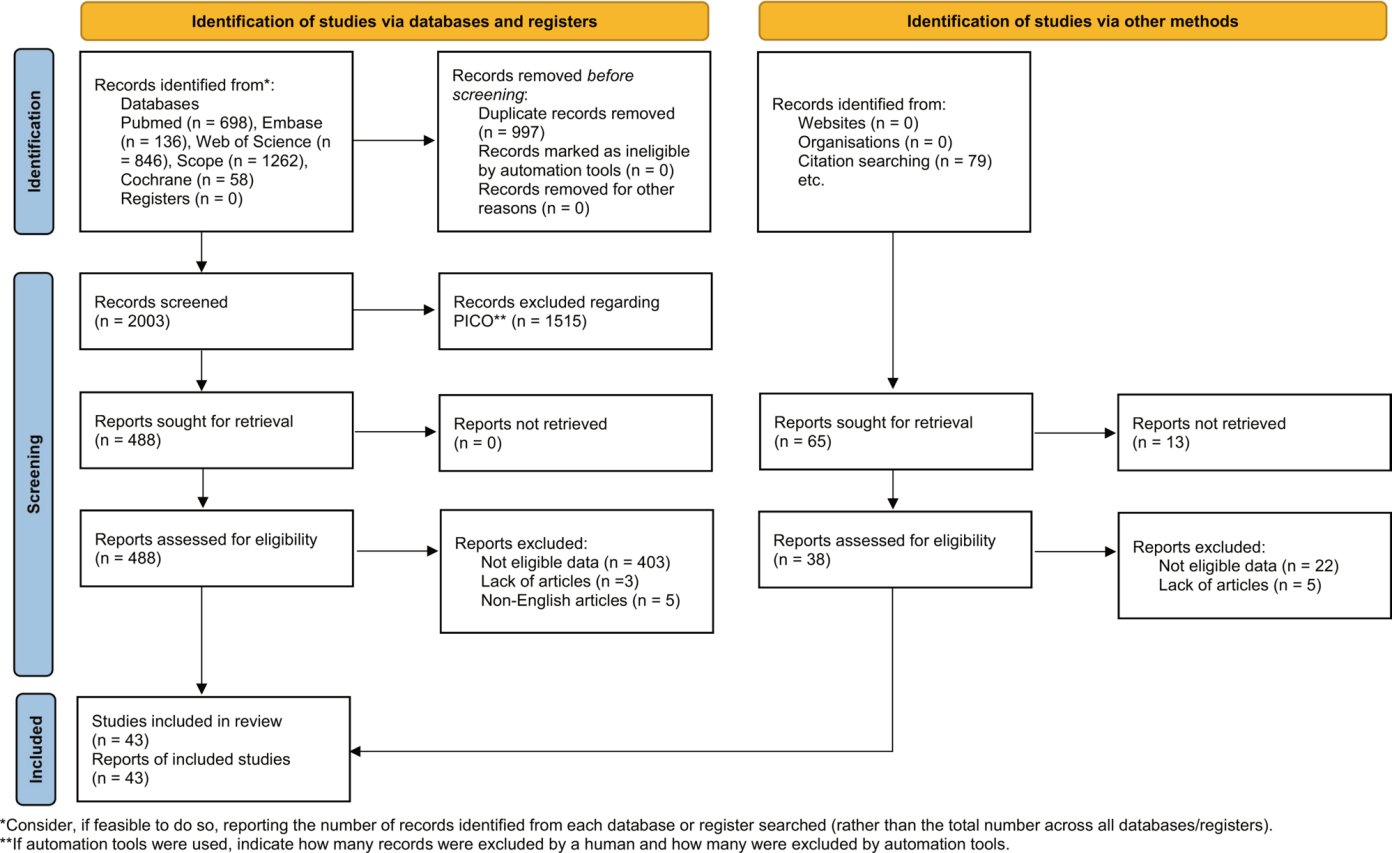
PRISMA 2020 flow diagram for new systematic reviews, which included searches of databases, registers and other sources

The PRISMA 2020 flow diagram for new systematic reviews, as depicted in Fig. 1, illustrates the process of the current systematic review. Out of 80 studies that met the inclusion criteria, 106 microRNAs were identified as potential hematologic tumor markers. However, due to the limited number of studies on most microRNAs, a comprehensive comparison via a systematic review and network meta-analysis was not feasible. Consequently, seven microRNAs with a substantial number of studies were selected for the network meta-analysis. The final selection comprised 43 studies [17–59] and seven prevalent microRNAs, namely, miR-150, 17, 20, 21, 23, 29, and 92.

The 28 case‒control studies and 15 cohort studies included in this review, as detailed in Table 1, were published between 2002 and 2022. The studies involved a total of 6008 CRC patients and 5341 healthy controls, with sample sizes ranging from 41 to 1302. The geographical distribution of the studies included ten from Europe and America, 25 from Asia, and eight from Africa. The quality assessments, also presented in Table 1, indicate a moderate to high overall quality for all studies, with CASP check scores exceeding nine for each.

**Table 1:** Characteristics of articles included in the network meta-analysis

First author and year	Country	Study design	Type of sample	Study period	CRC	non-CRC	Test	Sensitivity(%)	Specificity(%)	Accuracy(%)	Score
Ghareib 2020[10]	Egypt	case‒control	Serum	NR	48	48	miR-21	95.8	91.7	93.8	10.5
Yamada 2015[11]	Japan	cohort(validation)	Serum	2012–2014	136	52	miR-21	83.3	54.6	75.4	11
							miR-29	61.5	88.3	68.9	
Navarro 2020[12]	Spain	case‒control	Serum	2011–2017	27	45	miR-21	77.8	94	87.9	10
							miR-29	60	66.2	63.9	
							miR-92	68.9	89	81.5	
Bing 2012[13]	China	case‒control	Serum	2011	32	39	miR-21	97.5	74.4	84.8	10.5
Cheng 2017[14]	China	cohort(training)	Serum	2014–2016	60	60	miR-21	71.7	58.3	65	10.5
		cohort(validation)	Serum	2014–2016	80	80	miR-21	71.3	52.5	61.9	
							miR-17	85	45	65	
							miR-17	67.5	62.5	65	
							CEA	64.5	88.5	76.5	
							CEA	74.6	75	74.8	
Sabry 2018[15]	Egypt	case‒control	Serum	2013–2016	86	101	miR-21	91.4	95	93.3	11
Gang 2019[16]	China	case‒control	Serum	2017–2018	40	20	miR-21	88.9	83.3	87	9.5
							miR-210	88.9	72.2	83.3	
Basati 2014[17]	Iran	case‒control	Serum	2012–2013	40	40	miR-21	77	78	77.5	10
Guang-Hui 2013[18]	China	case‒control	Plasma	2006–2008	200	80	miR-21	65	85	70.7	11
							miR-92	65.5	82.5	70.4	
Wikberg 2018[19]	Sweden	cohort	Plasma	2010–2012	67	134	miR-21	80	78	78.7	10.5
Sarlinova 2014[20]	Slovak Republic	case‒control	Plasma	NR	71	80	miR-21	71.8	67.5	69.5	10.5
							miR-150	57.8	56.3	57	
							miR-221	71.8	68.8	70.2	
Bader 2020[21]	Egypt	cohort(validation)	Serum	NR	60	30	miR-21	80.7	100	87.1	9.5
Faltejskova 2016[22]	Czech Republic	cohort(training)	Serum	2010–2014	80	80	miR-21	82	70	76	10
	Czech Republic	cohort(validation)	Serum	2010–2014	203	100	miR-23	72.5	91	78.6	
	Czech Republic	cohort(training)	Serum	2010–2014	80	80	miR-23	89	77.5	83.3	
Hassan 2021[23]	Egypt	cohort	Serum	2018	52	20	miR-21	90.4	100	93.1	10
							miR-92	94.2	100	95.8	
Nonaka 2015[24]	Japan	case‒control	Serum	2011–2013	114	32	miR-21	54.7	84.4	61.2	11
Bastaminejad 2017[25]	Iran	case‒control	Serum	2014–2015	40	40	miR-21	86.1	73	79.5	10.5
Farouk 2020[26]	Egypt	case‒control	Serum	2017–2019	35	35	miR-21	82.9	97.1	90	10
							miR-23	82.9	91.4	87.1	
Sazanov 2016[27]	Russia	case‒control	Plasma	NR	31	34	miR-21	85	65	74.5	9.5
Shaohua 2018[28]	China	case‒control	Serum	2013–2014	107	120	miR-21	90.7	78.3	84.1	11
Xiaoya 2013[29]	Germany	case‒control	Plasma	2003–2007	80	144	miR-20	53.6	69.9	64.1	11
							miR-21	51.7	80.7	70.3	
							miR-29	30.5	90.4	69	
							miR-92	68.2	49.4	56.1	
Xihan 2020[30]	China	case‒control	Serum	2016	80	50	miR-21	90.6	86.2	88.9	10
							CEA	85.7	84.9	85.4	
Toiyama 2013[31]	Japan	cohort(validation)	Serum	2005–2010	200	53	miR-21	91.9	81.1	89.6	11
		cohort(training)	Serum	2005–2010	12	12	miR-21	82.8	90.6	86.7	
Yuntao 2021[32]	China	cohort	EVs	NR	100	35	miR-23	89.9	74.3	85.9	9
Kanaan 2012[33]	US	cohort(training)	Plasma	NR	30	30	miR-21	70	86	78	9
		cohort(validation)	Plasma	NR	20	20	miR-21	78	90	84	
Elshafei 2017[34]	Egypt	case‒control	Serum	NR	64	27	miR-92	84.4	100	89	9.5
Zaki 2022[35]	Egypt	case‒control	Serum	2016–2018	54	15	CA199	57.4	93.3	65.2	9
							CEA	66.7	80	69.6	
							miR-92	98.1	93.9	97.1	
Ng 2010[36]	China	case‒control	Plasma	NR	130	75	miR-17	59	84	68.1	10.5
							miR-92	80	93	84.8	
Giráldez 2013[37]	Spain	cohort(training)	Plasma	2009–2010	41	20	miR-19	93	75	87.1	10
		cohort(validation)	Plasma	2009–2010	82	53	miR-19	78.6	77.4	78.1	
Pi-Yueh 2016[38]	China	cohort(training)	Plasma	2012–2013	62	62	miR-92	76	77	76.5	10.5
		cohort(validation)	Plasma	2012–2013	153	121	miR-92	90.3	78	84.9	
Ying 2019[39]	China	case‒control	Serum	NR	148	68	miR-92	81.8	95.6	86.1	10.5
Zhaohui 2010[40]	China	case‒control	Plasma	NR	100	59	miR-29	69	89.1	76.5	10
							miR-92	84	71.2	79.3	
Berta 2019[41]	Spain	cohort(training)	EVs	NR	19	22	miR-21	68.8	67.7	68.3	9
		cohort(validation)	EVs	NR	19	22	miR-92	82.2	80.4	81.3	
							miR-29	87.4	90.5	87.8	
							miR-23	75.4	75.8	75.6	
							miR-20	90.6	84.3	87.7	
							miR-17	89.8	84.7	87.4	
							miR-150	93.6	89.9	91.9	
Basati 2015[42]	Iran	case‒control	Serum	2011–2012	50	55	miR-29	77	75	76	9.5
Leping 2015[43]	China	case‒control	Plasma	2013–2014	200	400	miR-29	61.4	72.5	68.8	11.5
Kawata 2014[44]	Japan	case‒control	Serum	2003–2004	88	11	CEA	30.7	100	38.4	10
							CA199	16	100	25.3	
							miR-150	55.7	100	60.6	
							miR-21	61.4	90.9	64.7	
							miR-23	92	100	92.9	
Karimi 2018[45]	Iran	case‒control	EVs	NR	25	13	miR-23	89	100	92.8	9
Zekri 2016[46]	Egypt	cohort(validation)	Serum	2011–2012	100	24	miR-17	80	100	83.9	10
							miR-20	70	100	75.8	
Jie 2020[47]	China	cohort(training)	Plasma	2014–2015	60	60	miR-20	65	96	80.5	10.5
		cohort(validation)	Plasma	2014–2015	597	585	miR-20	42	95	68.2	
Qinglan 2018[48]	China	case‒control	Serum	NR	46	33	miR-20	100	60	83.3	9.5
Wangyang 2015[49]	China	case‒control	Plasma	2007–2008	100	79	miR-20	46	73.4	58.1	10
Xiangxiang 2018[50]	China	cohort(training)	Plasma	NR	40	40	miR-20	68	92	80	10.5
		cohort(validation)	Plasma	NR	50	50	miR-20	60	92	76	
Holmström 2004[51]	Finland	case‒control	Serum	NR	28	161	CEA	55	66	64.4	9
							CA199	36	71	65.8	
Yajing 2019[52]	China	case‒control	EVs	2017–2018	165	155	miR-150	76	59	67.8	11

CRC: colorectal cancer; EVs: serum extracellular vesicles; NR: not reported. The scoring system based on the CASP checklist (specified for diagnostic studies) was applied for all studies.

### Pairwise meta-analysis

A traditional pairwise meta-analysis was conducted to estimate the odds ratio (OR) and 95% confidence interval (CI) for sensitivity and specificity. The summary results are presented in Table 2. The heterogeneity analysis results indicated that all I^2^ values exceeded 50%, necessitating the use of a random effects model for the Bayesian network meta-analysis.

**Table 2:** Traditional paired meta-analysis of the sensitivity and specificity of individual microRNAs

Test	Sensitivity			Specificity		
	estimate OR	95% CI	I^2^	estimate OR	95% CI	I^2^
CEA	0.65	0.48–0.78	92.31	0.81	0.70–0.89	76.14
miR-150	0.71	0.53–0.85	89.37	0.79	0.48–0.94	84.70
miR-17	0.73	0.58–0.84	82.08	0.69	0.51–0.83	89.07
miR-20	0.71	0.48–0.86	93.58	0.87	0.76–0.93	96.06
miR-21	0.81	0.76–0.86	87.86	0.82	0.77–0.87	82.80
miR-23	0.85	0.79–0.90	80.45	0.85	0.78–0.90	68.99
miR-29	0.64	0.51–0.75	87.61	0.83	0.74–0.89	86.49
miR-92	0.83	0.76–0.88	86.96	0.88	0.78–0.94	93.55

OR: odds ratio; CI: confidence interval

### Network geometry

**Fig. 2:**
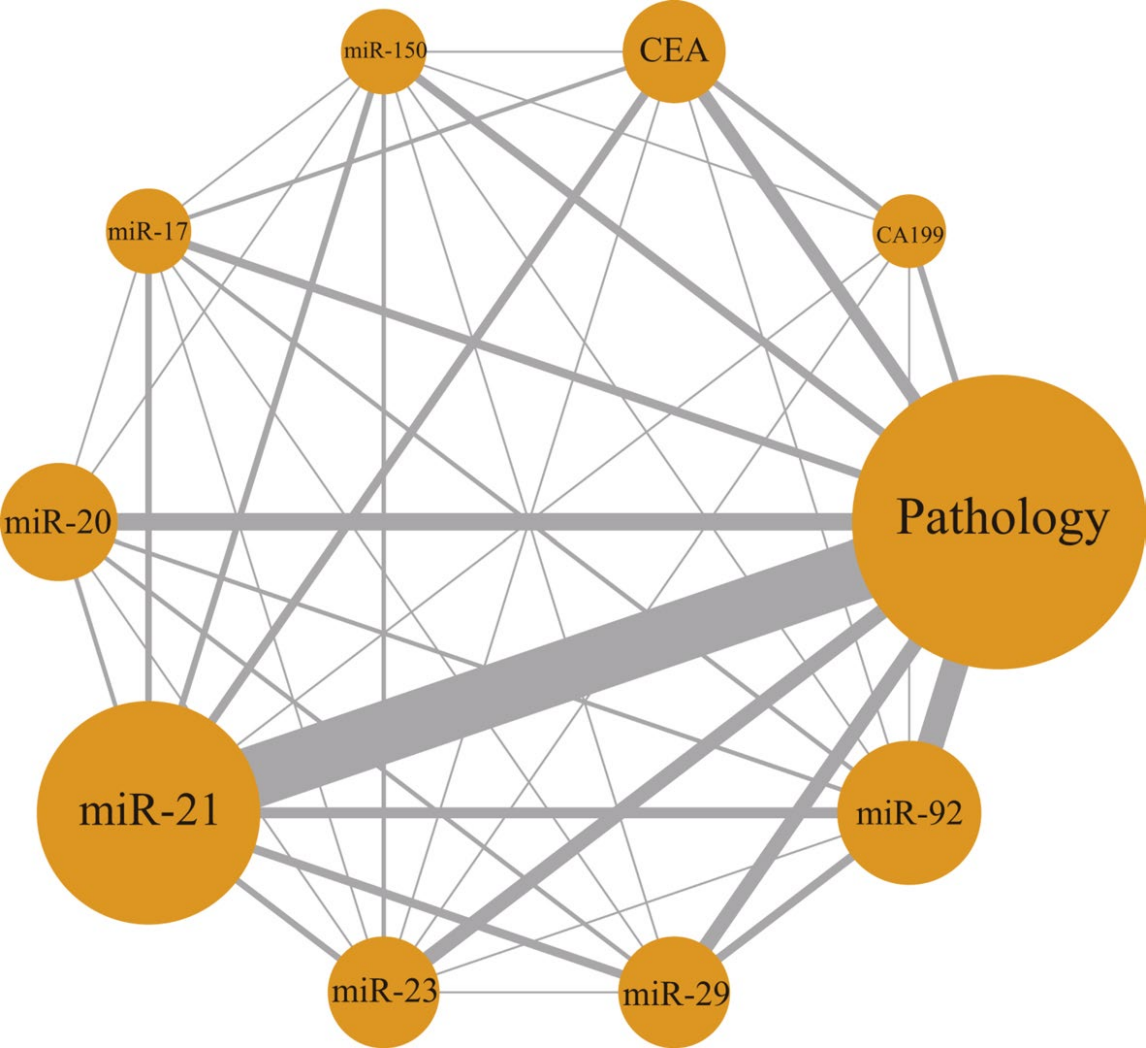
Evidence network plot of the diagnostic value of pathology and 7 different blood biomarker tests

Figure 2 illustrates the network structure, reflecting the relationships between the different marker studies. The size of the nodes corresponds to the number of studies included in the final analysis. Direct comparisons are indicated by solid lines between nodes, while the thickness and depth of the colors represent the number of studies compared between the two methods.

**Fig. 3:**
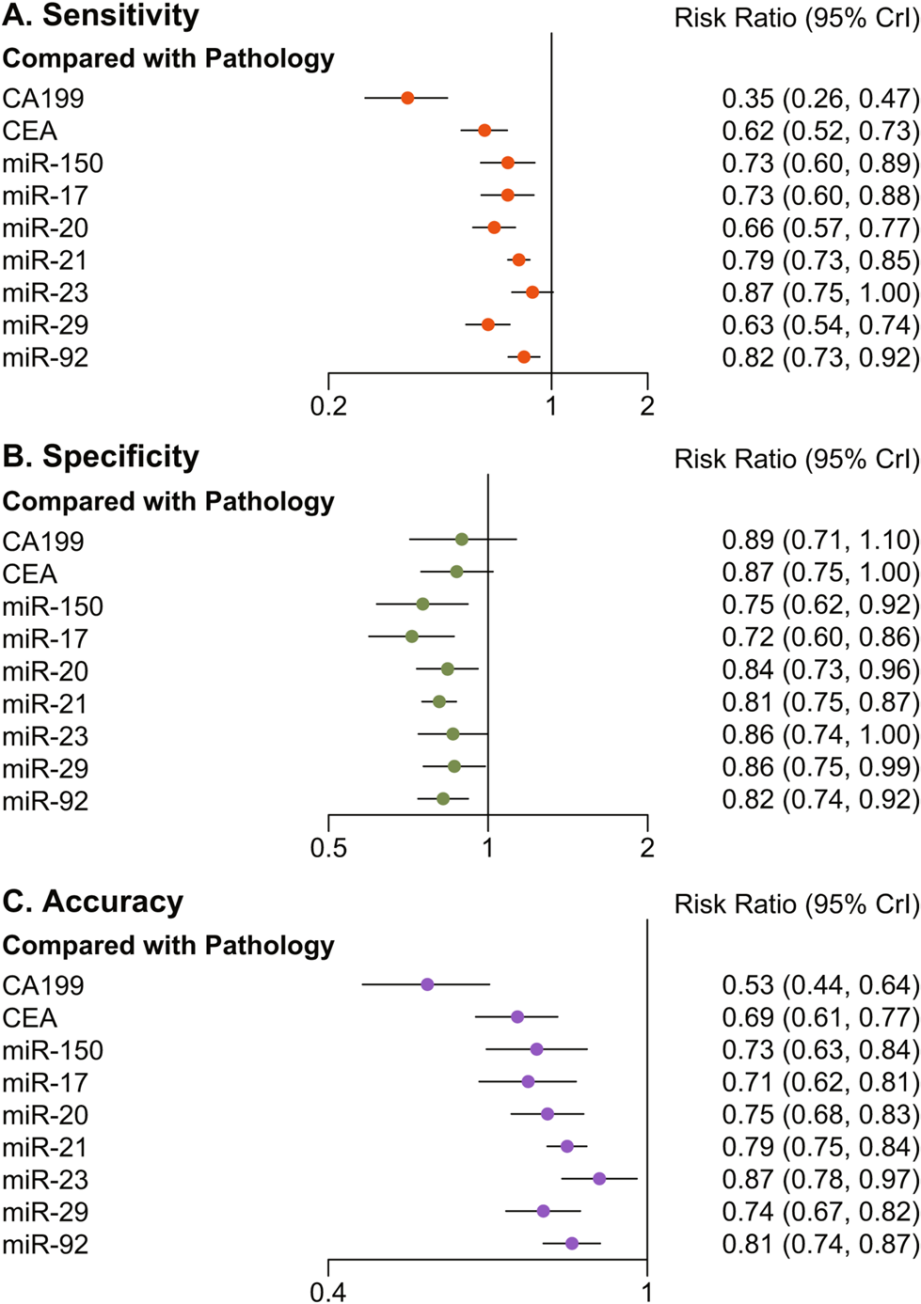
Forest plots of diagnostic sensitivity, specificity and accuracy of 7 miRNAs compared to pathological diagnosis. (**A**) Sensitivity, (**B**) Specificity, (**C**) Accuracy

### Sensitivity

Figure 3A illustrates that when benchmarked against postoperative pathological results, miR-29 exhibited the lowest diagnostic sensitivity among the seven peripheral circulating microRNA indicators, with a relative risk (RR) of 0.35 and a 95% confidence interval (CI) of 0.26–0.47. Conversely, miR-23 demonstrated the highest diagnostic sensitivity, with an RR of 0.87 and a 95% CI of 0.75-1.00, closely mirroring the postoperative pathological results.

Table 3 reveals that, in comparison to CA199 and CEA, commonly used clinical tumor indicators, all seven microRNA indicators displayed superior sensitivity to CA199, with miR-21, miR-23, and miR-92 outperforming CEA. miR-20 and miR-29 were less sensitive than miR-21, miR-23, and miR-92. A node-splitting analysis was conducted to evaluate the discrepancy between indirect and direct comparisons across all modalities. This analysis revealed inconsistencies in some comparisons (p value < 0.05), including those between miR-23 and CA199, CEA and miR-17, and miR-21 and CEA (Supplementary Fig. 1). These controversial comparisons are labeled in Table 3.

**Table 3:** Relative effects and 95% confidence intervals of all pairwise panels for sensitivity, specificity and accuracy based on the Bayesian network meta-analysis method

**Sensitivity**										
	Pathology	CA199	CEA	miR-150	miR-17	miR-20	miR-21	miR-23	miR-29	miR-92
**Pathology**	1									
CA199	0.35 (0.26, 0.47)	1								
CEA	0.62 (0.52, 0.73)	**1.7 (1.3, 2.4)**	1							
miR-150	0.73 (0.60, 0.89)	**2.1 (1.5, 2.9)**	1.2 (0.92, 1.5)	1						
miR-17	0.73 (0.60, 0.88)	**2.1 (1.5, 2.9)**	1.2 (0.94, 1.5)	1.0 (0.77, 1.3)	1					
miR-20	0.66 (0.57, 0.77)	**1.9 (1.4, 2.6)**	1.1 (0.86, 1.3)	0.91 (0.71, 1.2)	0.91 (0.72, 1.2)	1				
miR-21	0.79 (0.73, 0.85)	**2.2 (1.7, 3.0)**	1.3 (1.1, 1.5)*	1.1 (0.88, 1.3)	1.1 (0.89, 1.3)	**1.2 (1.0, 1.4)**	1			
miR-23	0.87 (0.75, 1.0)	2.5 (1.8, 3.4)*	1.4 (1.1, 1.8)*	1.2 (0.95, 1.5)	1.2 (0.95, 1.5)*	**1.3 (1.1, 1.6)**	1.1 (0.94, 1.3)	1		
miR-29	0.63 (0.54, 0.74)	**1.8 (1.3, 2.5)**	1.0 (0.82, 1.3)	0.87 (0.68, 1.1)	0.87 (0.68, 1.1)	0.96 (0.77, 1.2)	**0.80 (0.68, 0.95)**	**0.73 (0.59, 0.90)**	1	
miR-92	0.82 (0.73, 0.92)	**2.3 (1.7, 3.2)**	**1.3 (1.1, 1.6)**	1.1 (0.90, 1.4)	1.1 (0.92, 1.4)	**1.2 (1.0, 1.5)**	1.0 (0.91, 1.2)	0.94 (0.78, 1.1)	**1.3 (1.1, 1.6)**	1
**Specificity**										
	Pathology	CA199	CEA	miR-150	miR-17	miR-20	miR-21	miR-23	miR-29	miR-92
Pathology	1									
CA199	**0.89 (0.71, 1.1)**	1								
CEA	0.87 (0.75, 1.0)	0.98 (0.76, 1.2)	1							
miR-150	0.75 (0.62, 0.92)	0.84 (0.62, 1.1)	0.86 (0.67, 1.1)	1						
miR-17	0.72 (0.60, 0.86)	0.80 (0.60, 1.1)	**0.82 (0.66, 1.0)**	0.95 (0.73, 1.2)	1					
miR-20	0.84 (0.73, 0.96)	0.94 (0.72, 1.2)	0.96 (0.78, 1.2)	1.1 (0.88, 1.4)	1.2 (0.94, 1.5)	1				
miR-21	0.81 (0.75, 0.87)	0.91 (0.71, 1.1)*	0.93 (0.79, 1.1)	1.1 (0.88, 1.3)	1.1 (0.93, 1.4)	0.96 (0.83, 1.1)	1			
miR-23	0.86 (0.74, 1.0)	0.96 (0.73, 1.3)	0.98 (0.79, 1.2)	1.1 (0.90, 1.5)	1.2 (0.95, 1.5)	1.0 (0.84, 1.2)	1.1 (0.90, 1.3)	1		
miR-29	0.86 (0.75, 0.99)	0.97 (0.74, 1.3)	0.99 (0.81, 1.2)	1.1 (0.91, 1.4)	1.2 (0.96, 1.5)	1.0 (0.86, 1.2)	1.1 (0.92, 1.2)	1.0 (0.83, 1.2)	1	
miR-92	0.82 (0.74, 0.92)	0.92 (0.72, 1.2)	0.94 (0.78, 1.1)	1.1 (0.88, 1.4)	1.1 (0.94, 1.4)	0.98 (0.83, 1.2)	1.0 (0.90, 1.2)	0.96 (0.80, 1.1)	0.95 (0.81, 1.1)	1
**Accuracy**										
	Pathology	CA199	CEA	miR-150	miR-17	miR-20	miR-21	miR-23	miR-29	miR-92
Pathology	1									
CA199	0.53 (0.44, 0.63)	1								
CEA	0.69 (0.61, 0.77)	**1.3 (1.1, 1.6)**	1							
miR-150	0.73 (0.63, 0.84)	**1.4 (1.1, 1.7)**	1.1 (0.89, 1.3)	1						
miR-17	0.71 (0.62, 0.82)	**1.3 (1.1, 1.7)**	1.0 (0.87, 1.2)	0.97 (0.80, 1.2)	1					
miR-20	0.75 (0.68, 0.83)	**1.4 (1.2, 1.7)**	1.1 (0.93, 1.3)	1.0 (0.87, 1.2)	1.1 (0.89, 1.3)	1				
miR-21	0.79 (0.75, 0.84)	**1.5 (1.2, 1.8)**	1.2 (1.0, 1.3)*	1.1 (0.94, 1.3)	1.1 (0.97, 1.3)	1.1 (0.94, 1.2)	1			
miR-23	0.87 (0.78, 0.97)	1.6 (1.3, 2.0)*	1.3 (1.1, 1.5)*	**1.2 (1.0, 1.4)**	1.2 (1.0, 1.5)*	**1.2 (1.0, 1.3)**	1.1 (0.98, 1.2)	1		
miR-29	0.74 (0.67, 0.83)	**1.4 (1.1, 1.7)**	1.1 (0.92, 1.3)	1.0 (0.86, 1.2)	1.0 (0.88, 1.2)	0.99 (0.86, 1.1)	**0.93 (0.83, 1.0)**	**0.85 (0.73, 0.99)**	1	
miR-92	0.81 (0.74, 0.87)	**1.5 (1.3, 1.8)**	**1.2 (1.0, 1.3)**	1.1 (0.94, 1.3)	1.1 (0.97, 1.3)	1.1 (0.94, 1.2)	1.0 (0.92, 1.1)	0.92 (0.81, 1.1)	1.1 (0.96, 1.2)	1

* The analysis by node splitting revealed inconsistent results between indirect and direct comparisons

**Fig. 4:**
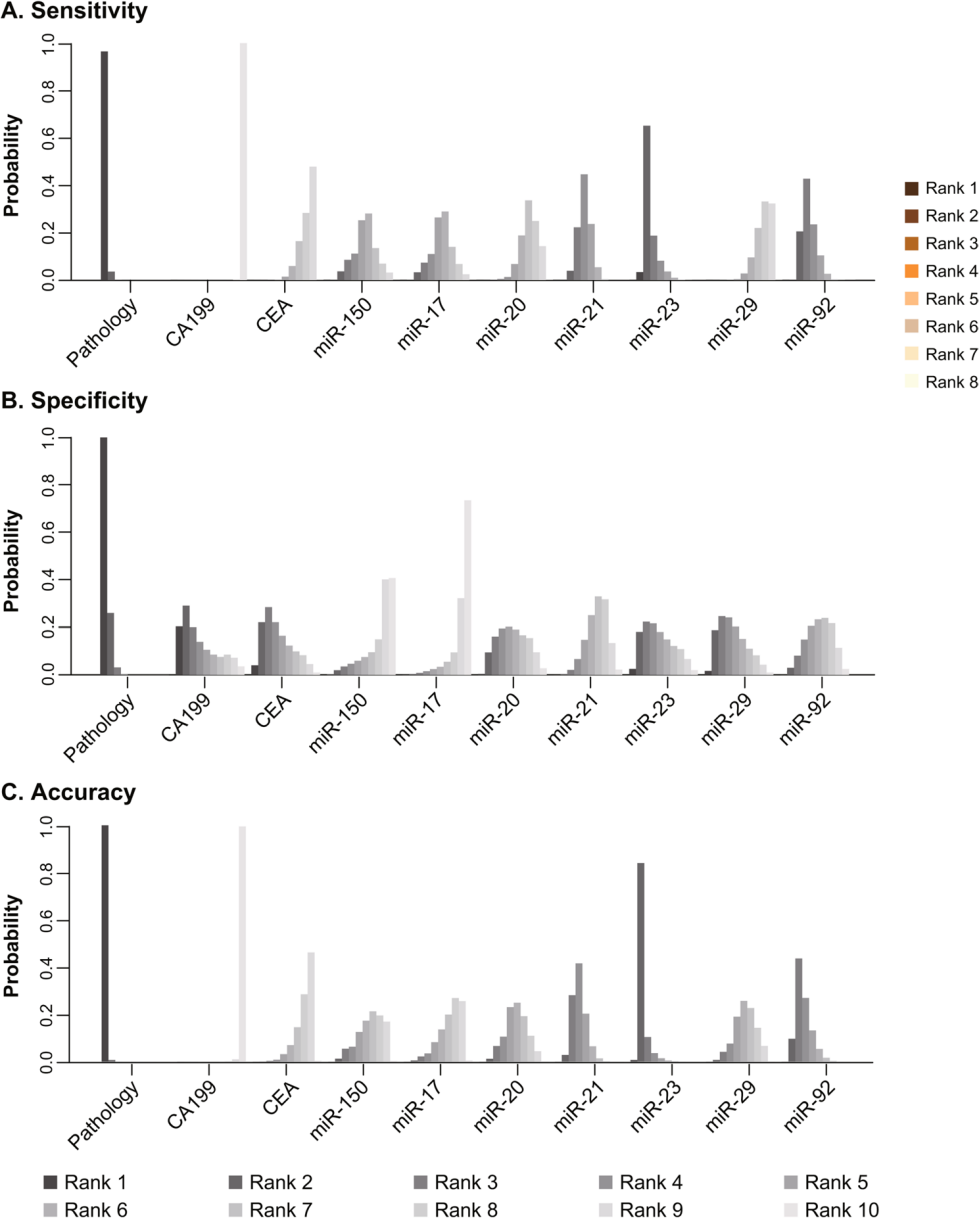
Estimated rank probability of 7-miRNA sensitivity, specificity, and accuracy. (**A**) Sensitivity, (**B**) Specificity, (**C**) Accuracy

The diagnostic sensitivities of the seven microRNAs, in descending order, were miR-23, 92, 21, 17, 150, 20, and 29, as depicted in the rankograms in Fig. 4A and the ranking table in Table 4.

**Table 4:** Ranking of sensitivity, specificity and accuracy of different miRNAs in diagnosing CRC

**Sensitivity**										
	Rank1	Rank2	Rank3	Rank4	Rank5	Rank6	Rank7	Rank8	Rank9	Rank10
**Postoperative pathology**	**0.9647**	0.0351	0.0002	0	0	0	0	0	0	0
CA199	0	0	0	0	0	0	0	0	0.0005	**0.9994**
CEA	0	0.0001	0.0005	0.0015	0.0146	0.0594	0.1649	0.2804	**0.4784**	0.0002
miR-150	0.0009	0.0372	0.0851	0.1114	0.2555	**0.2768**	0.1336	0.0678	0.0319	0
miR-17	0.0006	0.0326	0.0713	0.1077	**0.2638**	0.2907	0.1423	0.0663	0.0247	0
miR-20	0	0.0009	0.0045	0.0126	0.0682	0.1874	**0.3305**	0.2514	0.1445	0.0001
miR-21	0	0.0377	0.2211	**0.4478**	0.2364	0.0531	0.0038	0.0002	0	0
**miR-23**	0.0333	**0.6518**	0.1873	0.082	0.0342	0.0102	0.0012	0.0002	0	0
miR-29	0	0.0001	0.0007	0.0029	0.0259	0.0954	0.2212	**0.3334**	0.32	0.0003
miR-92	0.0005	0.2047	**0.4294**	0.2341	0.1014	0.027	0.0026	0.0003	0	0
**Specificity**										
	Rank1	Rank2	Rank3	Rank4	Rank5	Rank6	Rank7	Rank8	Rank9	Rank10
Postoperative pathology	**0.771**	0.2042	0.0234	0.0014	0	0	0	0	0	0
CA199	0.1597	**0.2237**	0.1565	0.107	0.0817	0.065	0.0591	0.0667	0.0534	0.0273
CEA	0.0319	0.1689	**0.2193**	0.1708	0.1287	0.0971	0.0768	0.0623	0.0361	0.0082
miR-150	0.0019	0.0156	0.0269	0.0363	0.0455	0.0576	0.0741	0.1145	**0.3086**	0.319
miR-17	0.0002	0.0025	0.0068	0.0128	0.0186	0.0273	0.0413	0.0754	0.25	**0.5651**
miR-20	0.0035	0.0737	0.1231	0.1508	**0.1569**	0.1452	0.1284	0.1236	0.0746	0.0202
miR-21	0	0.0031	0.0157	0.0514	0.1137	0.1949	**0.2561**	0.2435	0.105	0.0167
miR-23	0.0193	0.1411	0.1739	0.1644	0.1398	**0.1123**	0.0944	0.0859	0.0524	0.0167
**miR-29**	0.0124	0.144	0.1886	**0.1884**	0.1565	0.1156	0.0861	0.0653	0.0343	0.009
miR-92	0.0003	0.0231	0.066	0.1167	0.1586	0.1851	0.1837	**0.1629**	0.0857	0.018
**Accuracy**										
	Rank1	Rank2	Rank3	Rank4	Rank5	Rank6	Rank7	Rank8	Rank9	Rank10
Postoperative pathology	**0.9929**	0.0071	0	0	0	0	0	0	0	0
CA199	0	0	0	0	0	0.0001	0.0004	0.0016	0.0088	**0.989**
CEA	0	0.0004	0.0025	0.0072	0.0301	0.0703	0.1459	0.2779	**0.4622**	0.0037
miR-150	0	0.0121	0.0541	0.0663	0.1254	0.1682	**0.2101**	0.1949	0.1665	0.0024
miR-17	0	0.0049	0.0235	0.035	0.0823	0.1336	0.1965	**0.2687**	0.2513	0.0041
miR-20	0	0.012	0.065	0.1038	**0.225**	0.2509	0.1903	0.1083	0.0445	0.0003
miR-21	0	0.0271	0.2744	**0.4115**	0.2064	0.0642	0.0146	0.0018	0.0001	0
**miR-23**	0.0071	**0.8327**	0.1029	0.0363	0.0144	0.005	0.0014	0.0003	0	0
miR-29	0	0.0066	0.04	0.0774	0.1844	**0.2555**	0.2257	0.1437	0.0662	0.0005
miR-92	0	0.0972	**0.4375**	0.2624	0.1322	0.0522	0.0151	0.0029	0.0004	0

### Specificity

Figure 3B indicates that miR-17 had the lowest diagnostic specificity among the seven peripheral blood circulating microRNA indicators, with an RR of 0.72 and a 95% CI of 0.60–0.86. miR-29 demonstrated the highest specificity, with an RR of 0.86 and a 95% CI of 0.75–0.99.

Compared to CA199 and CEA, miR-17 was less specific than CEA, while the remaining microRNAs did not exhibit statistically significant differences in specificity. Table 3 shows no significant difference in diagnostic specificity among the microRNAs. Node-split analysis revealed inconsistency between indirect and direct comparisons between miR-21 and CA199 (Supplementary Fig. 2).

The diagnostic specificity of the seven microRNAs, in descending order, were miR-29, 20, 23, 21, 92, 150, and 17, as depicted in the rankograms in Fig. 4B and the ranking table in Table 4.

### Accuracy

Figure 3C shows that miR-17 had the lowest diagnostic accuracy among the seven peripheral blood circulating microRNA indicators, with an RR of 0.71 and a 95% CI of 0.62–0.81. miR-23 demonstrated the highest accuracy, with an RR of 0.87 and a 95% CI of 0.78–0.97.

Table 3 reveals that, compared to CA199 and CEA, all seven microRNA indicators displayed superior accuracy to CA199, with miR-21, miR-23, and miR-92 outperforming CEA. In the comparison of diagnostic accuracy among microRNAs, miR-23 was superior to miR-150, miR-17, and miR-20, while miR-29 was inferior to miR-21 and miR-23. Node-split analysis revealed inconsistency between indirect and direct comparisons between miR-23 and CA199, CEA, and miR-17, as well as comparisons between miR-21 and CEA (Supplementary Fig. 3).

The diagnostic accuracy of the seven microRNAs, in descending order, were miR-23, 92, 21, 20, 29, 150, and 17, as depicted in the rankograms in Fig. 4C and the ranking table in Table 4.

### Publication bias

**Fig. 5:**
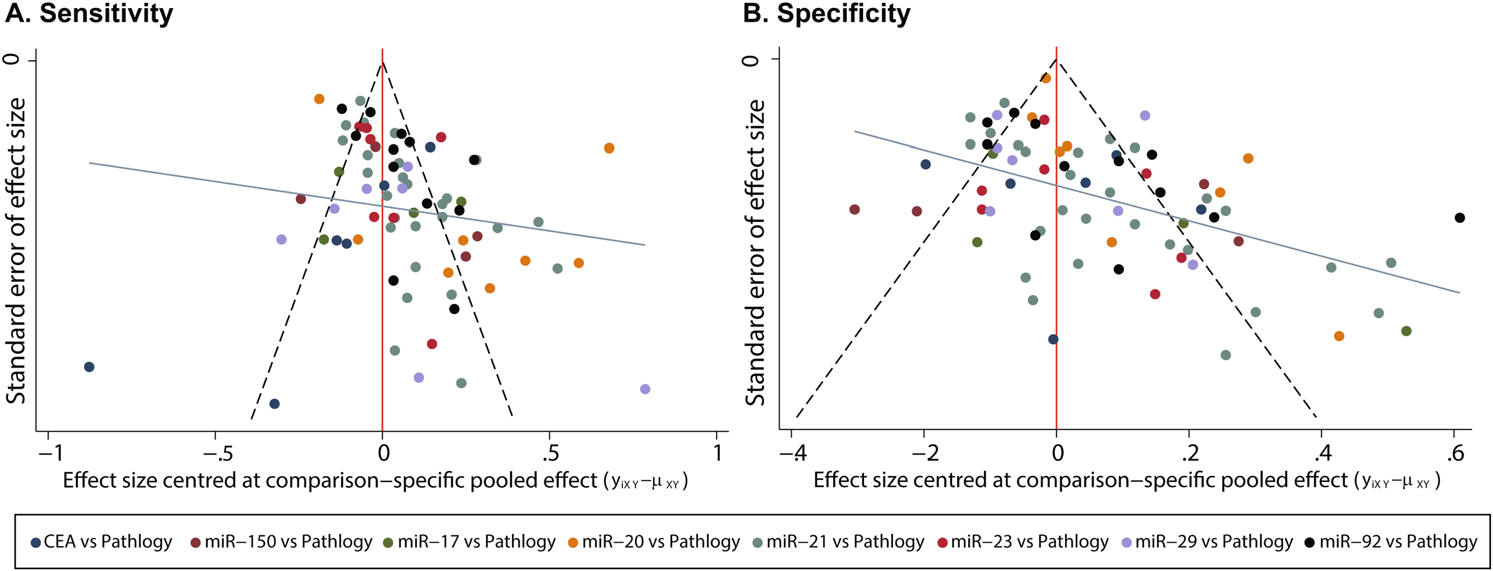
Comparative adjusted funnel plot for publication bias

To assess the publication bias of this study, a funnel plot was constructed. Figure 5a and b present the funnel plots for sensitivity and specificity, respectively. The relative symmetry of the plots suggests a minimal publication bias, which can be disregarded. Subsequently, Egger’s test (*p* = 0.241 for sensitivity and 0.188 for specificity) also support the view.

The gray line symbolizes the null hypothesis that the study-specific effect sizes are not different from the respective comparison-specific pooled effect estimates. The green line represents the regression line, with different colors corresponding to different comparisons.

## Discussion

Multidrug resistance in cancer makes the treatment increasingly difficult [3], so early identification of colorectal tumors is crucial in mitigating the mortality rates associated with colorectal cancer. Blood tumor markers are considered as straightforward, noninvasive, and readily available among the various diagnostic methods for colorectal cancer. Circulating miRNAs, capable of withstanding adverse physiological conditions such as extreme pH and temperature fluctuations and multiple freeze‒thaw cycles, have recently emerged as a promising tool for early colorectal tumor screening [60]. Given the broad spectrum of circulating miRNAs, each with differing sensitivity and specificity, a network meta-analysis was conducted to compare each circulating miRNA’s diagnostic pros and cons, correlating them with the commonly employed blood-based colorectal cancer biomarkers CA-199 and CEA.

miR-23 exhibited the highest sensitivity, and higher sensitivity reduces the likelihood of false negatives, thereby saving time in conducting more definitive diagnostic tests [61]. miR-29 demonstrated the highest specificity, and high specificity (high true-negative rate) can prevent significant psychological stress or additional diagnostic costs for the patient due to false-positive tests [61]. The accuracy of an assay, its ability to correctly distinguish between patients and healthy cases, is generally judged by its sensitivity and specificity. Our findings indicate that miR-23 has the highest accuracy and overall function compared to other markers. miR-23 is an oncomiR that inhibits the expression of pyruvate dehydrogenase kinase 4, which activates pyruvate dehydrogenase and oxidative phosphorylation to produce sufficient ATP for cell proliferation [62]. Wang et al. found that miR-23a promotes the migration and invasion of CRC cells and tumor stem cells by targeting the metastasis suppressor 1 (MTSS1) gene [63]. The expression of miR-23a was reported to be positively correlated with the clinical stage and infiltration depth of the tumor, and high expression levels of miR-23a in tissues were found to be a poor prognostic marker of cancer [64].

Given that a single tumor marker indicator cannot fulfill the screening needs in terms of detection sensitivity and specificity, the concurrent use of multiple blood tumor markers as a panel can enhance sensitivity and specificity. Referring to this network meta-study, the more studied circulating miRNAs with high diagnostic accuracy, such as miR-23, 21, 92, can be selected for panel composition, and pairing with traditional blood CEA assays to improve the panel’s specificity.

Next, how to integrate the newly identified miRNAs into the existing colorectal cancer screening program? Firstly, it is necessary to conduct multicenter studies with independent samples to validate the sensitivity, specificity, and predictive value of these miRNAs. After confirming their effectiveness, the screened miRNA tests can be used as standalone tests or in combination with existing tests (such as fecal occult blood test and colonoscopy). When used as an independent screening tool, miRNA tests can be used as a preliminary screening tool to identify high-risk individuals for further colonoscopy examination. When used as a combined screening tool, miRNA tests can be used in conjunction with existing tests like fecal occult blood test to improve the accuracy and coverage of screening.

Additionally, we have designed methods to compare the cost-effectiveness of miRNA testing with traditional screening methods to evaluate its economic feasibility. First, cost analysis will calculate the direct costs of miRNA testing (including reagents, equipment, and labor) compared to the costs of traditional screening methods. Next, benefit analysis will estimate the long-term benefits such as medical cost savings and improved quality of life due to early detection of colorectal cancer. Lastly, a cost-effectiveness model will be established using decision tree models [65] or Markov models [66] to compare the long-term health economic outcomes of different screening strategies. Factors to consider include cancer detection rates, treatment success rates, and changes in quality of life caused by screening. Finally, after confirming the effectiveness of the miRNA screening method, specific implementation plans will be formulated and this new strategy will be promoted through appropriate channels, including education and training, public awareness, and policy support.

Our analysis was constrained by the data of the included studies and the structure of the reported data. Initially, 79 articles were screened with a total of 105 miRNAs, but not all of them could be used in our analysis, so the 7 most studied miRNA metrics were streamlined based on the number of studies. In some articles where sensitivity and/or specificity were unavailable in the original article, sensitivity and specificity were indirectly derive using AUC plots (principle of maximum area under the curve), which may result in observed heterogeneity in pairwise meta-analyses and potentially affect the accuracy of network meta-analyses. Additionally, due to the limitations in the data of the included studies, this article did not perform subgroup analysis on colorectal cancer at different stages of development. Whether the diagnostic value of miRNA is universally applicable in early and advanced colorectal cancer requires further investigation. Last, although the sensitivity and specificity of miR-23 were high, the number of articles studying miR-23 was still needed to be improved, thus potentially leading to inconsistencies between the results of direct and indirect comparisons shown in the nodal analysis.

## Conclusions

In conclusion, circulating microRNAs have high diagnostic value for colorectal cancer, which is not inferior to traditional CEA and CA19-9. miR-23, 92, and 21 had high diagnostic value in terms of sensitivity, with sensitivities of 87%, 82%, and 79%, respectively, when combined. In terms of specificity, miR-29, 23, and 20 had high diagnostic value, and the specificity after combination was 86%, 86%, and 84%, respectively. Combining sensitivity and specificity, miR-23, 92, and 21 had high accuracies of 87%, 81%, and 79%, respectively. This systematic review and network meta-analysis may provide a reference basis for the clinical selection of circulating miRNAs as tumor biomarkers for the early detection of CRC and improved survival of CRC patients.

### Electronic supplementary material

Below is the link to the electronic supplementary material.

**Supplementary Figure1:** Node-splitting analysis of inconsistency for sensitivity.

**Supplementary Figure 2:** Node-splitting analysis of inconsistency for specificity.

**Supplementary Figure 3:** Node-splitting analysis of inconsistency for accuracy.

## Data Availability

The data used to support the findings of this study are available from the corresponding author upon request.

## Abbreviations

CASP: Critical Appraisal Skills Programme
RR: Risk ratio
CI: Confidence interval
INPLASY: International Platform of Registered Systematic Review and Meta-analysis Protocols
PRISMA: Preferred Reporting Items for Systematic Reviews and Meta-analysis
MeSH: Medical subject heading
ROC: Receiver operating characteristics
CRC: Colorectal cancer
EVs: Serum extracellular vesicles
NR: Not reported
OR: Odds ratio
