# Supplementary figures and images for: Comparison of the diagnostic value of various microRNAs in blood for colorectal cancer: a systematic review and network meta-analysis
Source: BMC Cancer. 2024 Jun 26;24:770. doi: 10.1186/s12885-024-12528-8 (PMC11209970; doi:10.1186/s12885-024-12528-8)

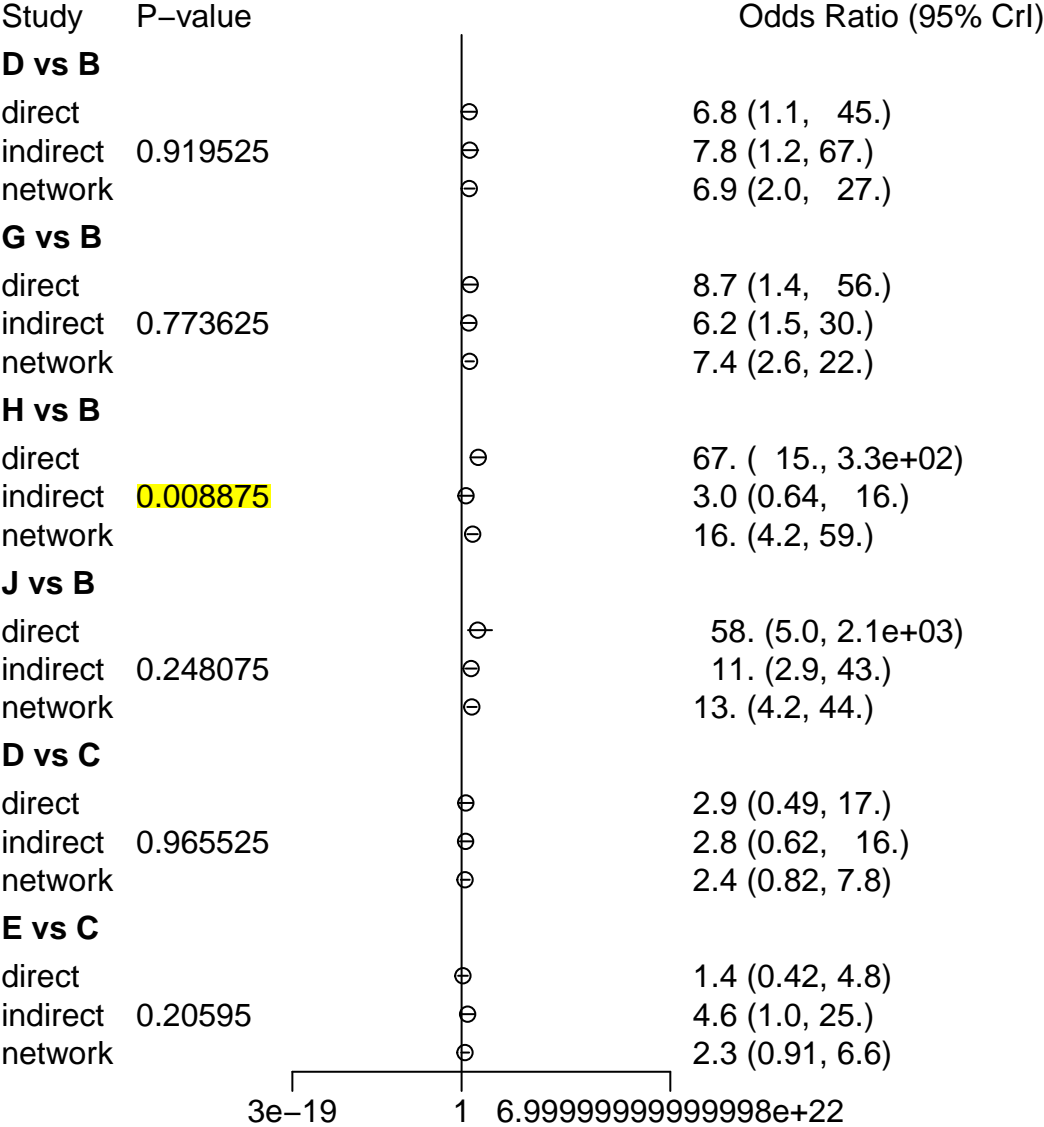

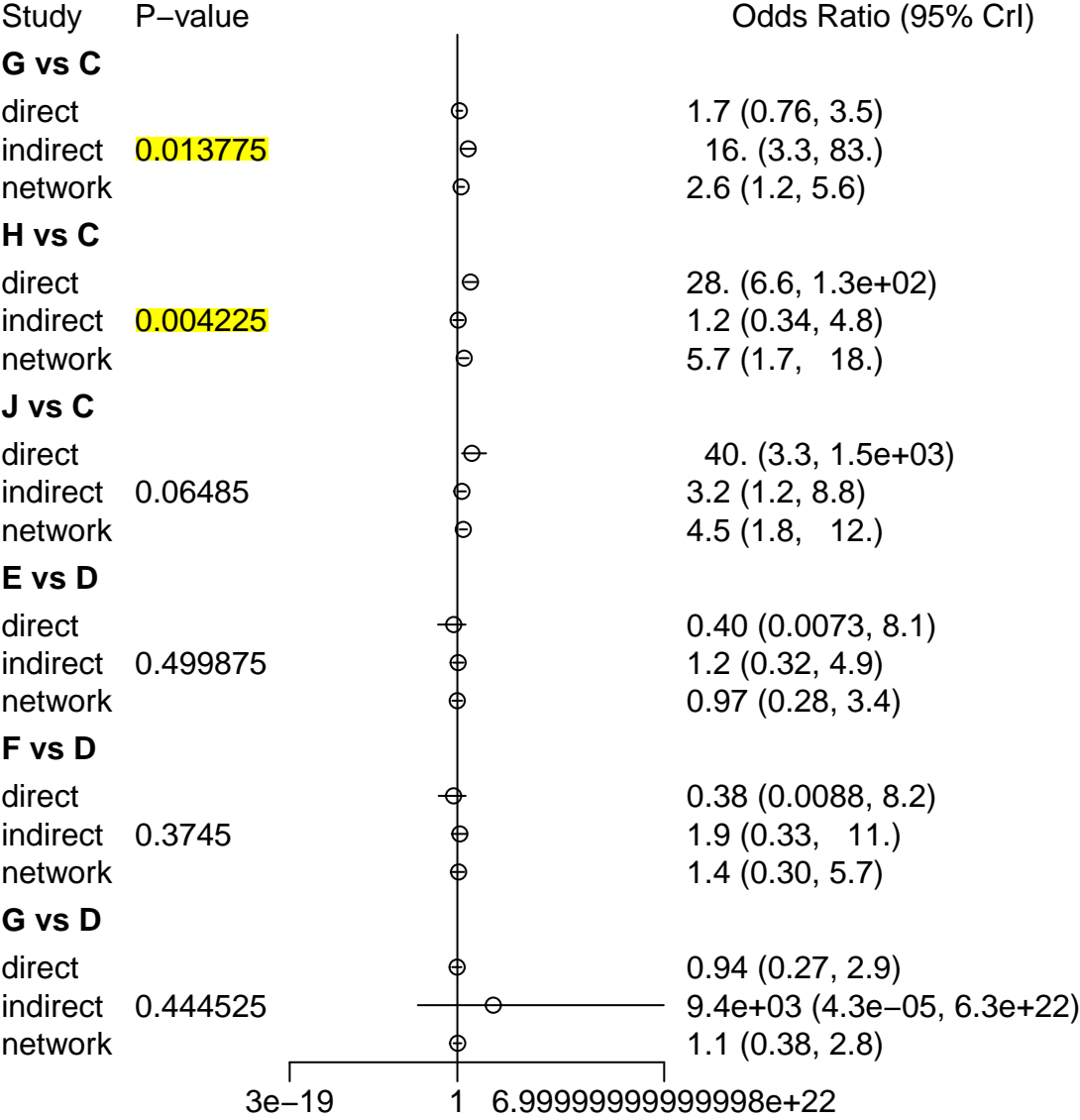

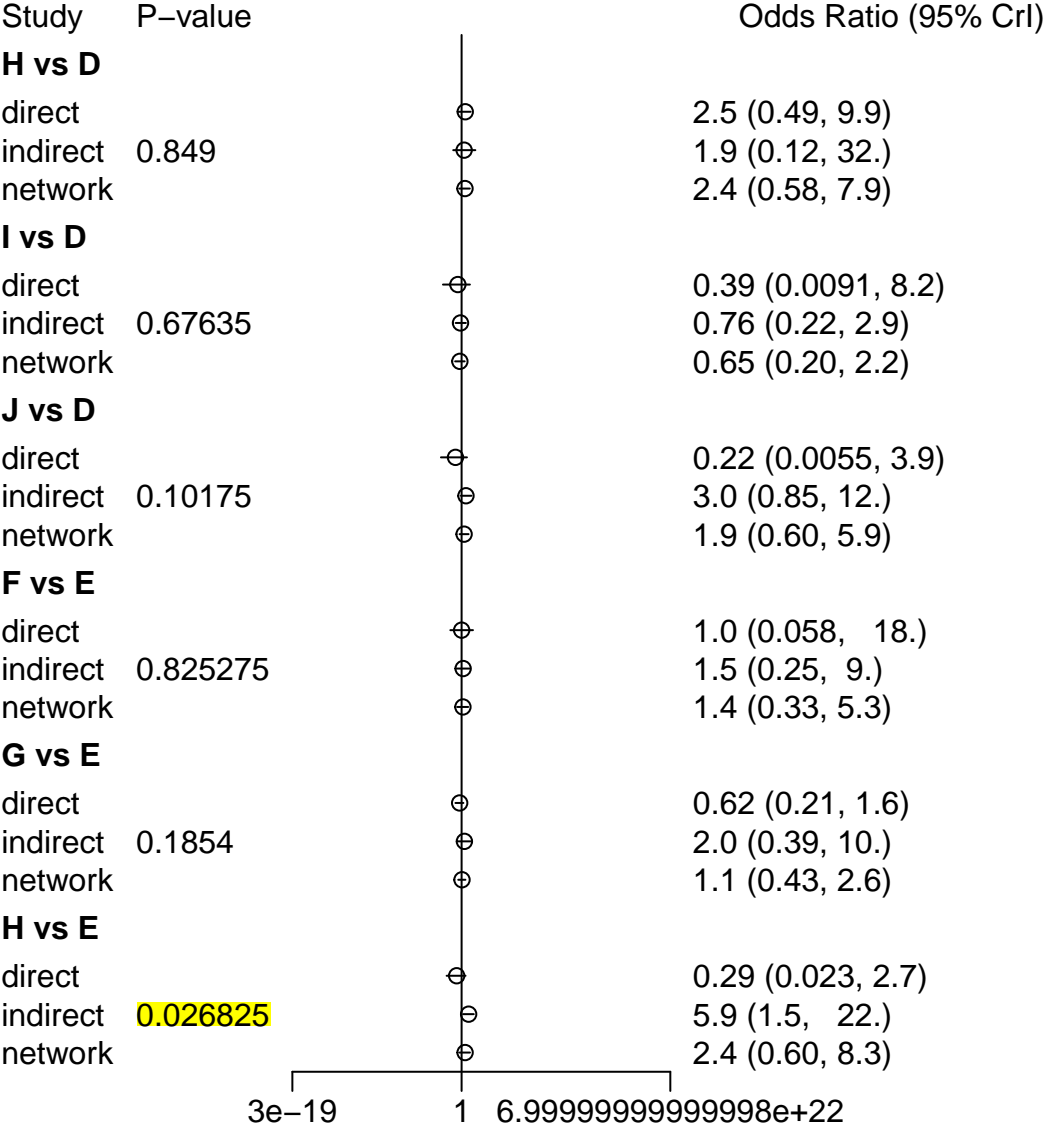

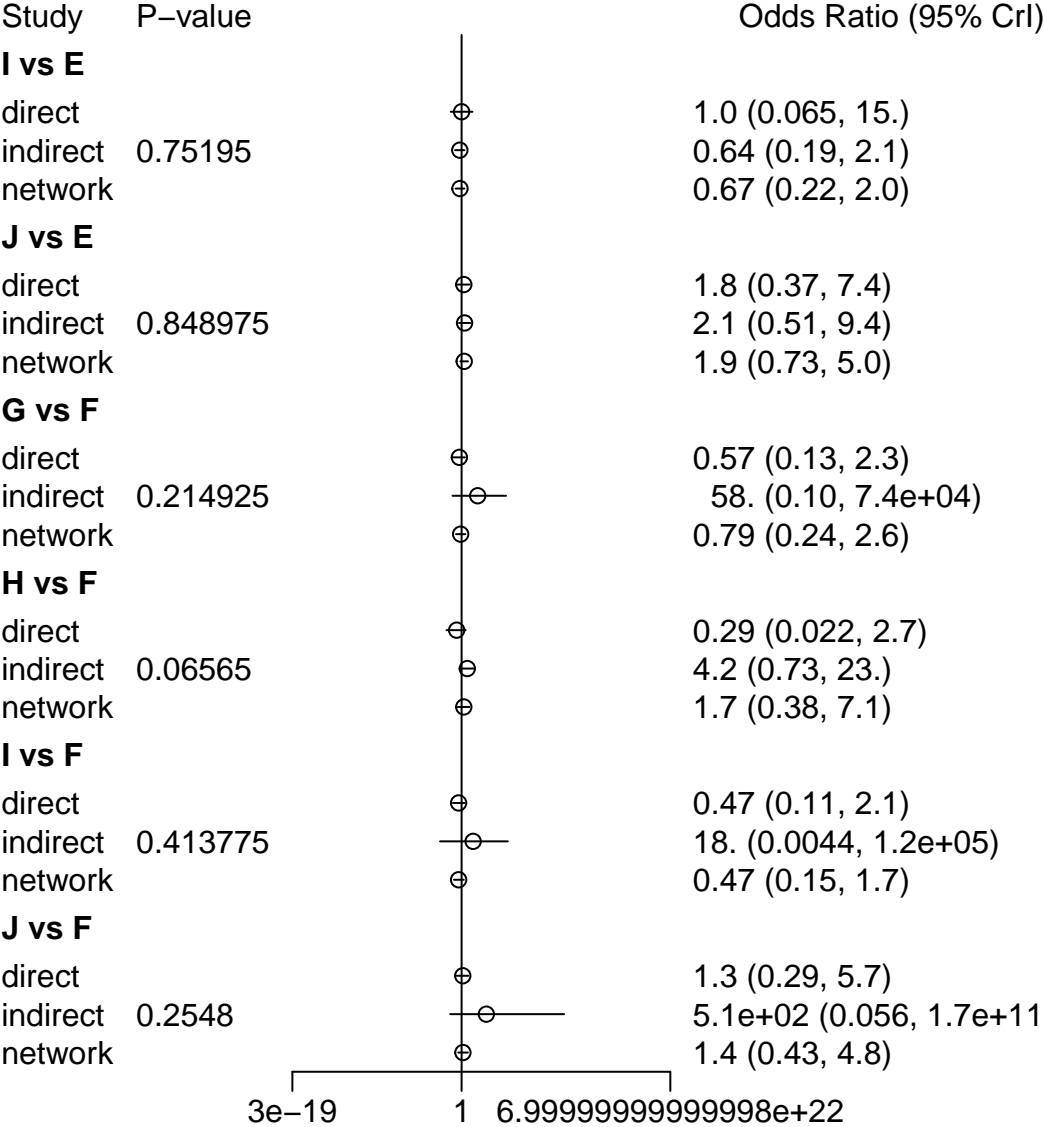

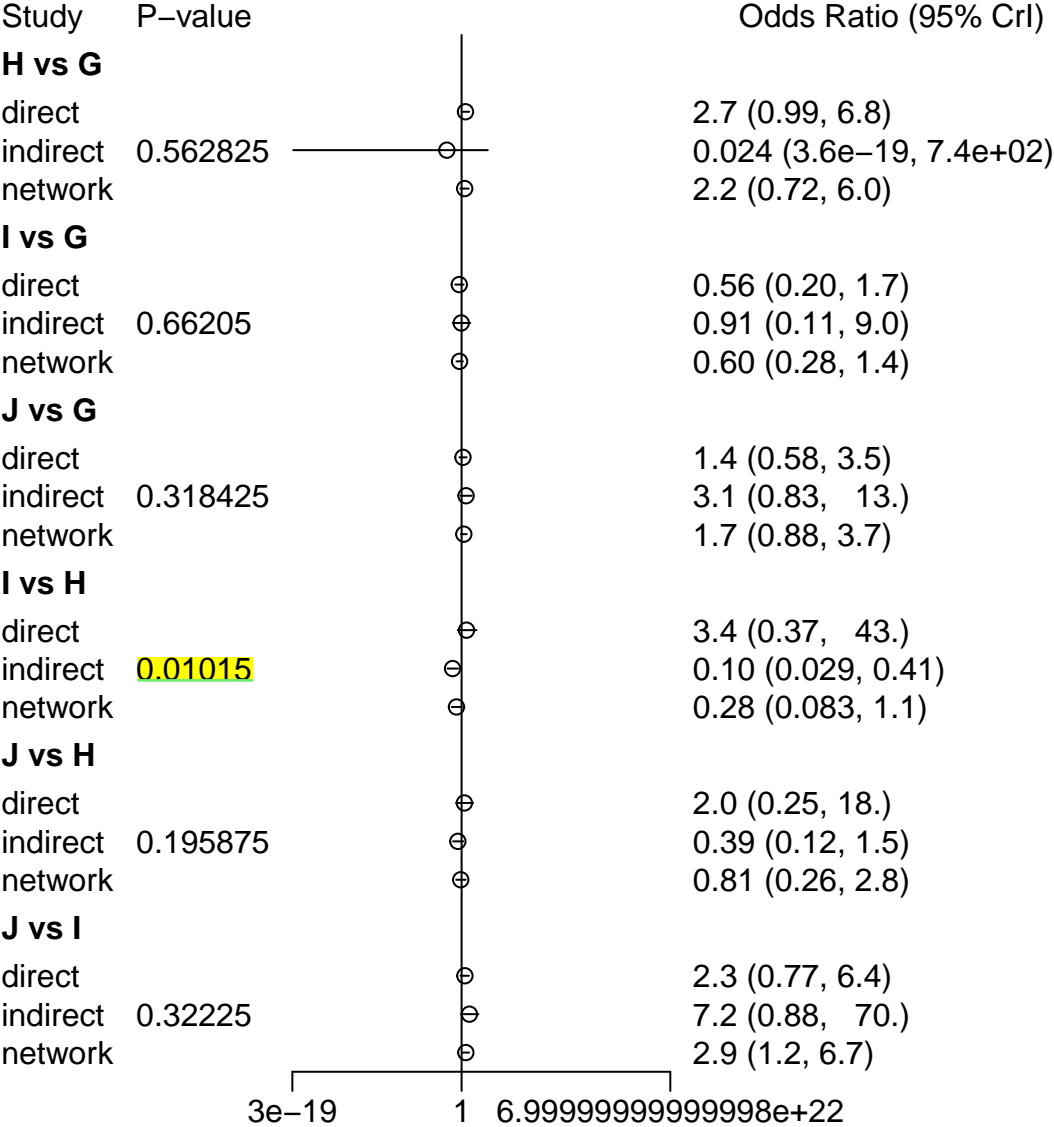

Supplement: Supplementary file 1 — Supplementary Figure1: Node-splitting analysis of inconsistency for sensitivity. [file 12885_2024_12528_MOESM1_ESM.pdf]

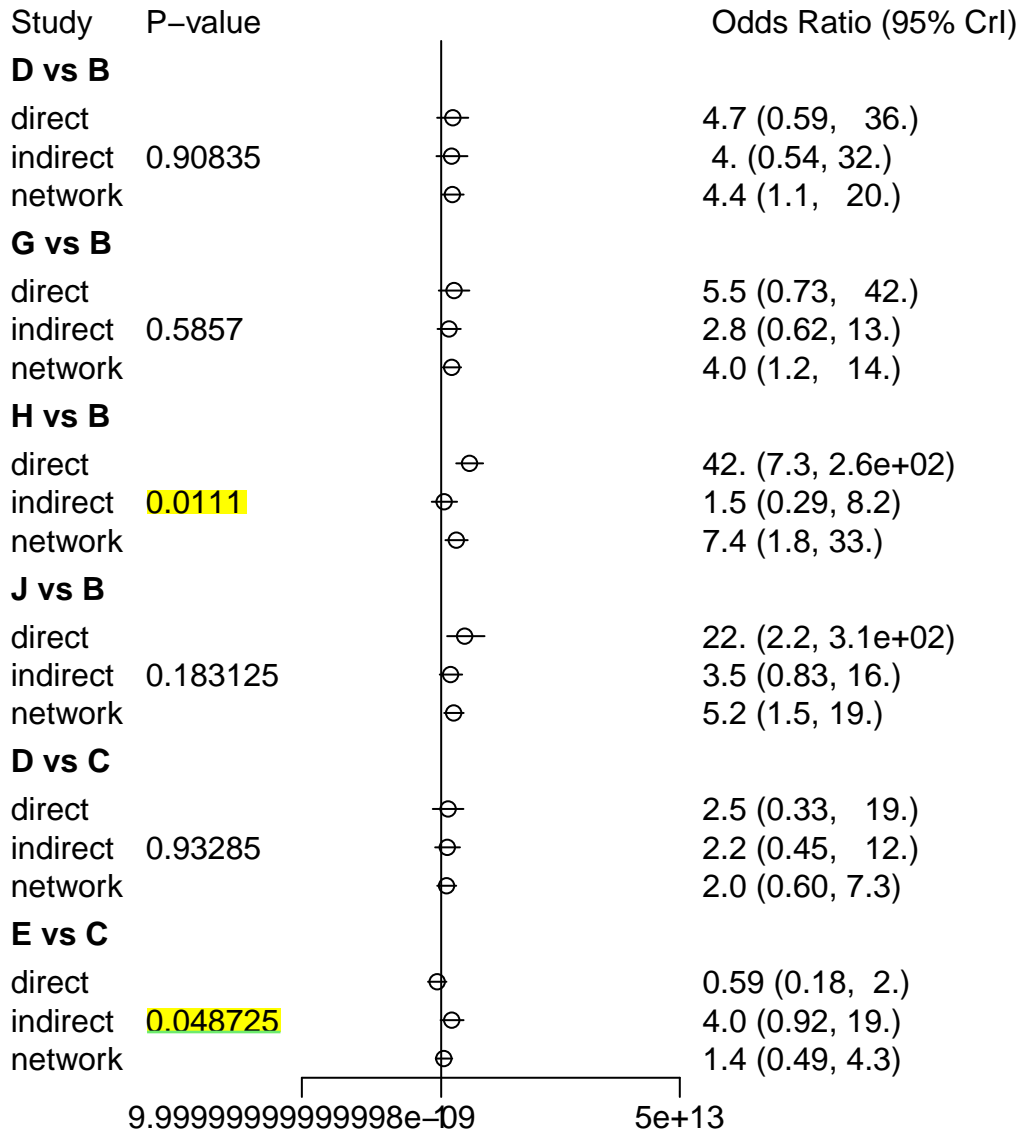

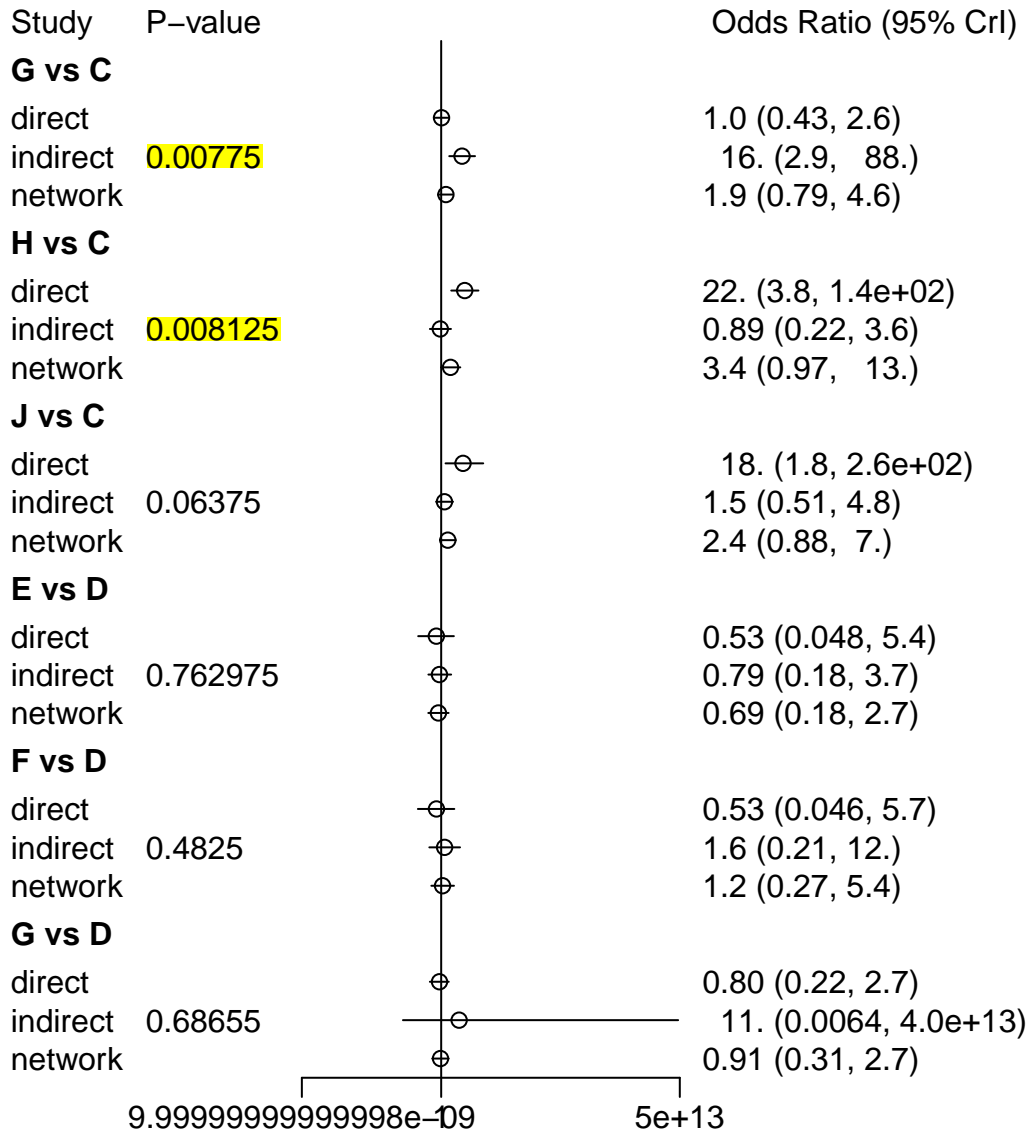

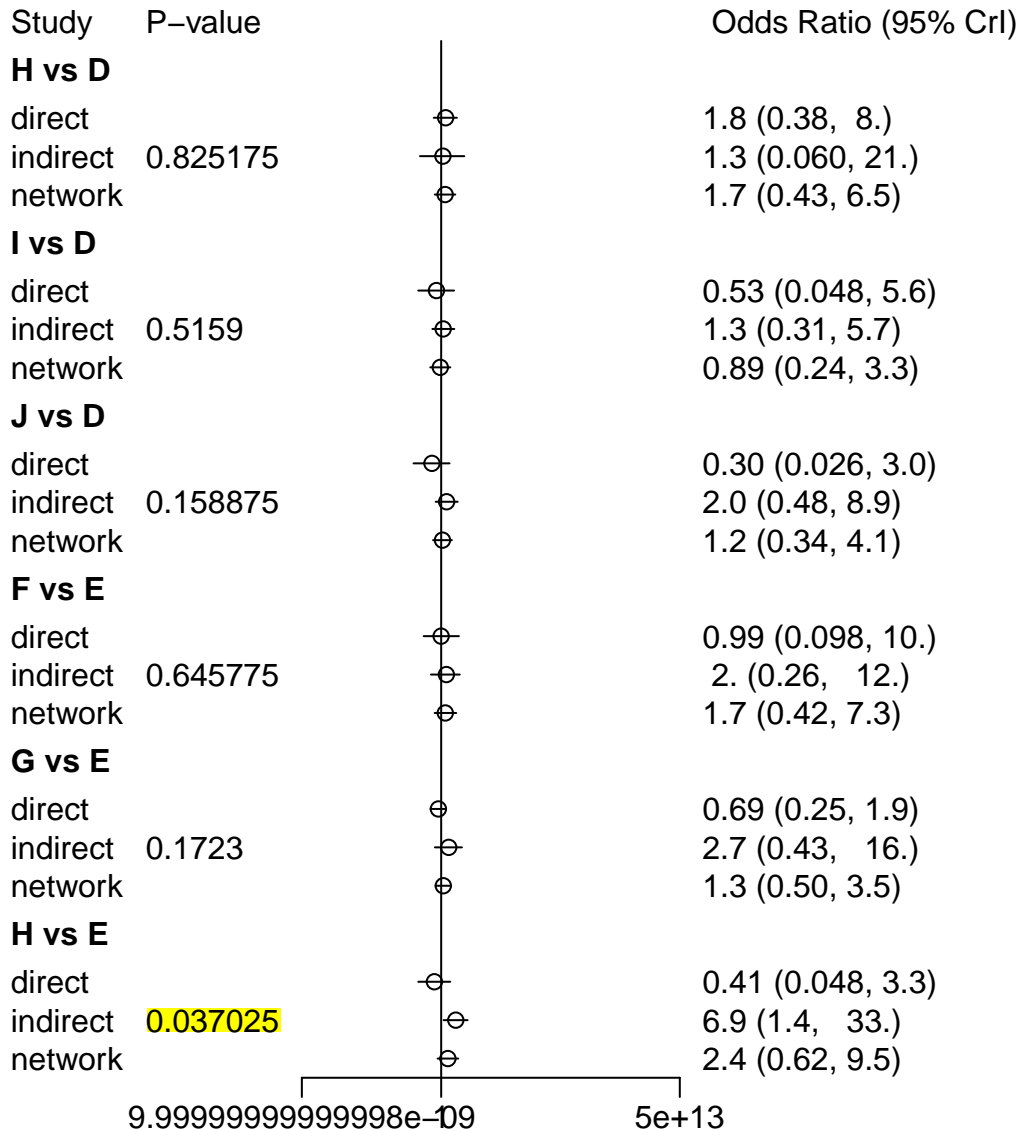

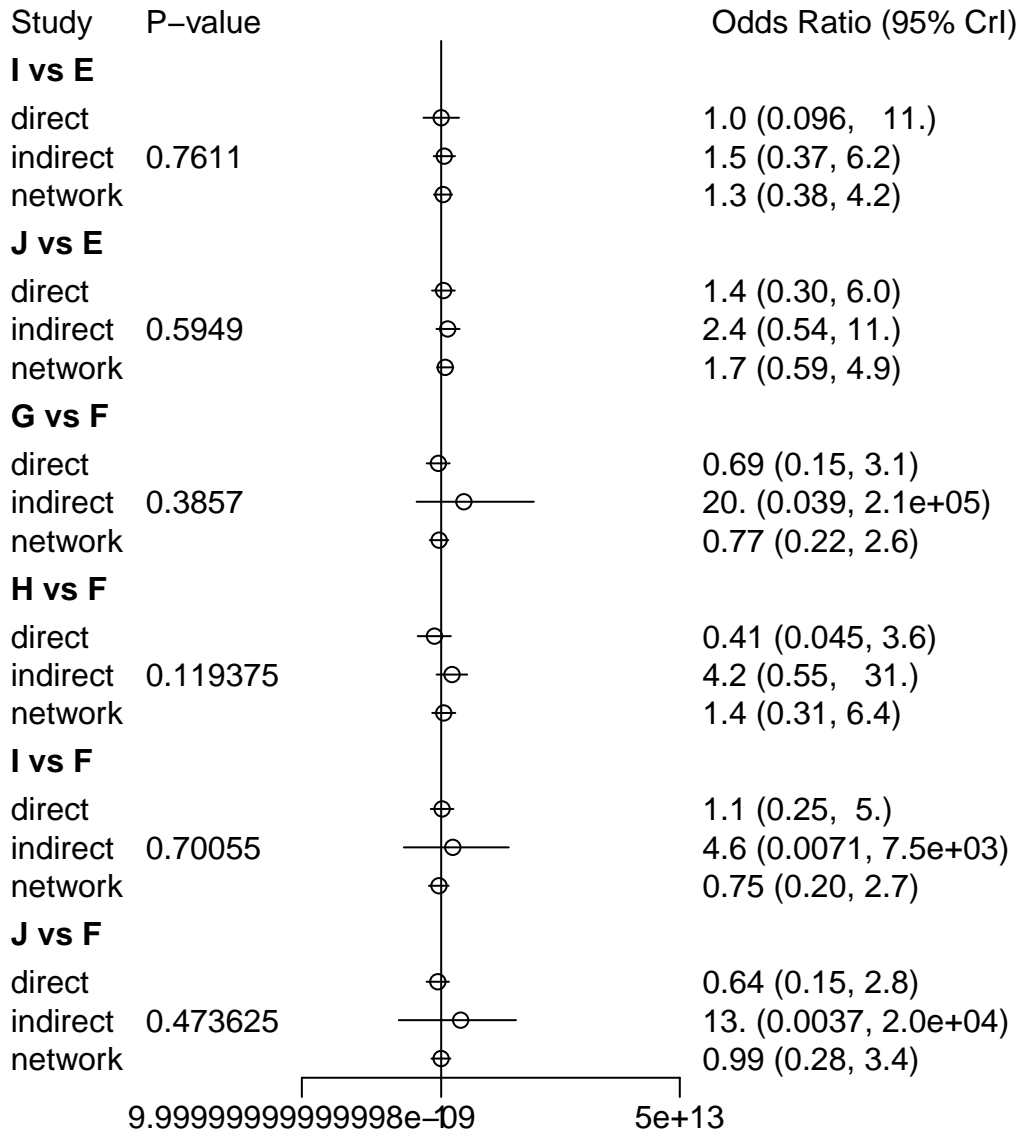

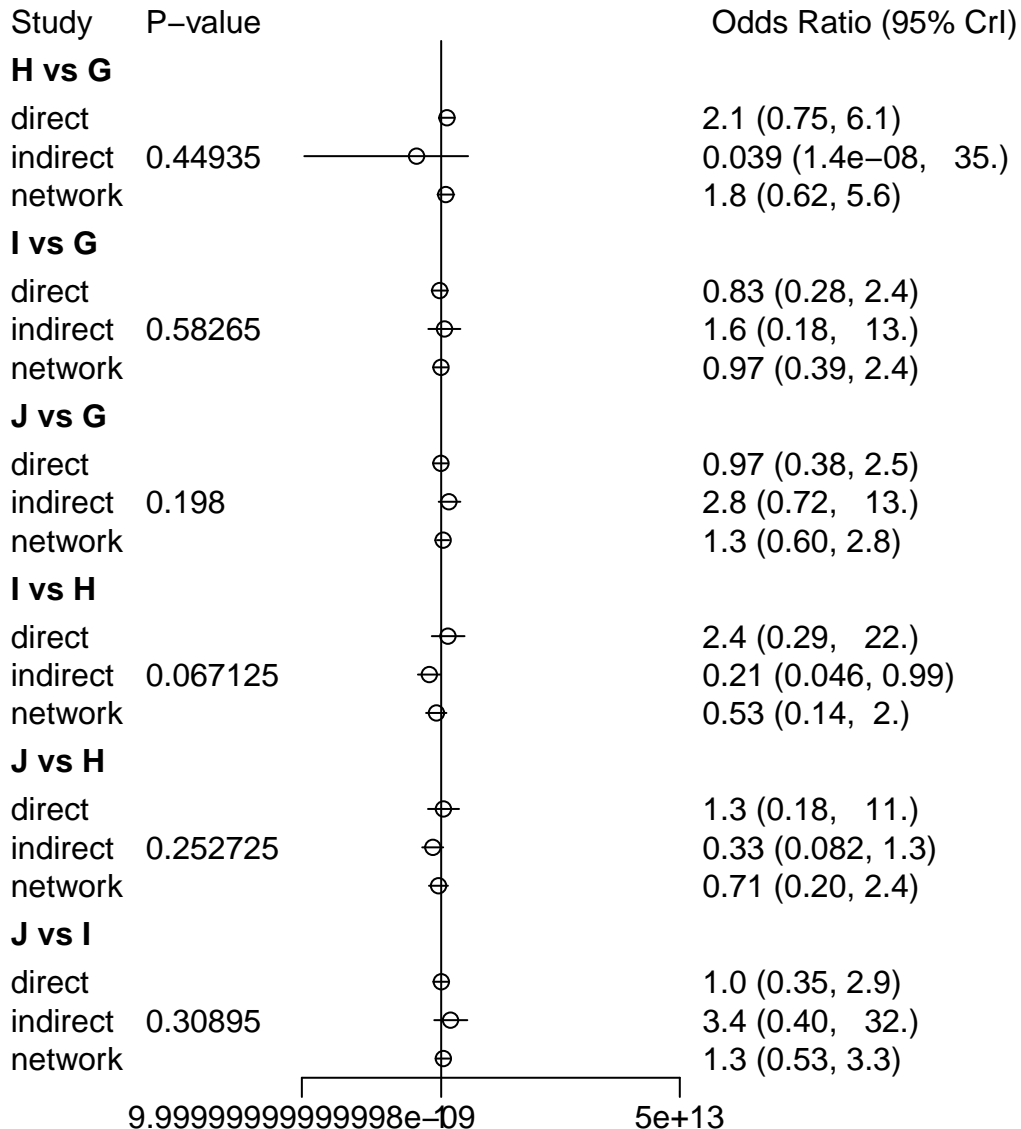

Supplement: Supplementary file 3 — Supplementary Figure 3: Node-splitting analysis of inconsistency for accuracy. [file 12885_2024_12528_MOESM3_ESM.pdf]
