# Supplementary material for: Comparison of the diagnostic value of various microRNAs in blood for colorectal cancer: a systematic review and network meta-analysis
Source: BMC Cancer. 2024 Jun 26;24:770. doi: 10.1186/s12885-024-12528-8 (PMC11209970; doi:10.1186/s12885-024-12528-8)
Supplement: Supplementary file 2 — Supplementary Figure 2: Node-splitting analysis of inconsistency for specificity. [file 12885_2024_12528_MOESM2_ESM.pdf]

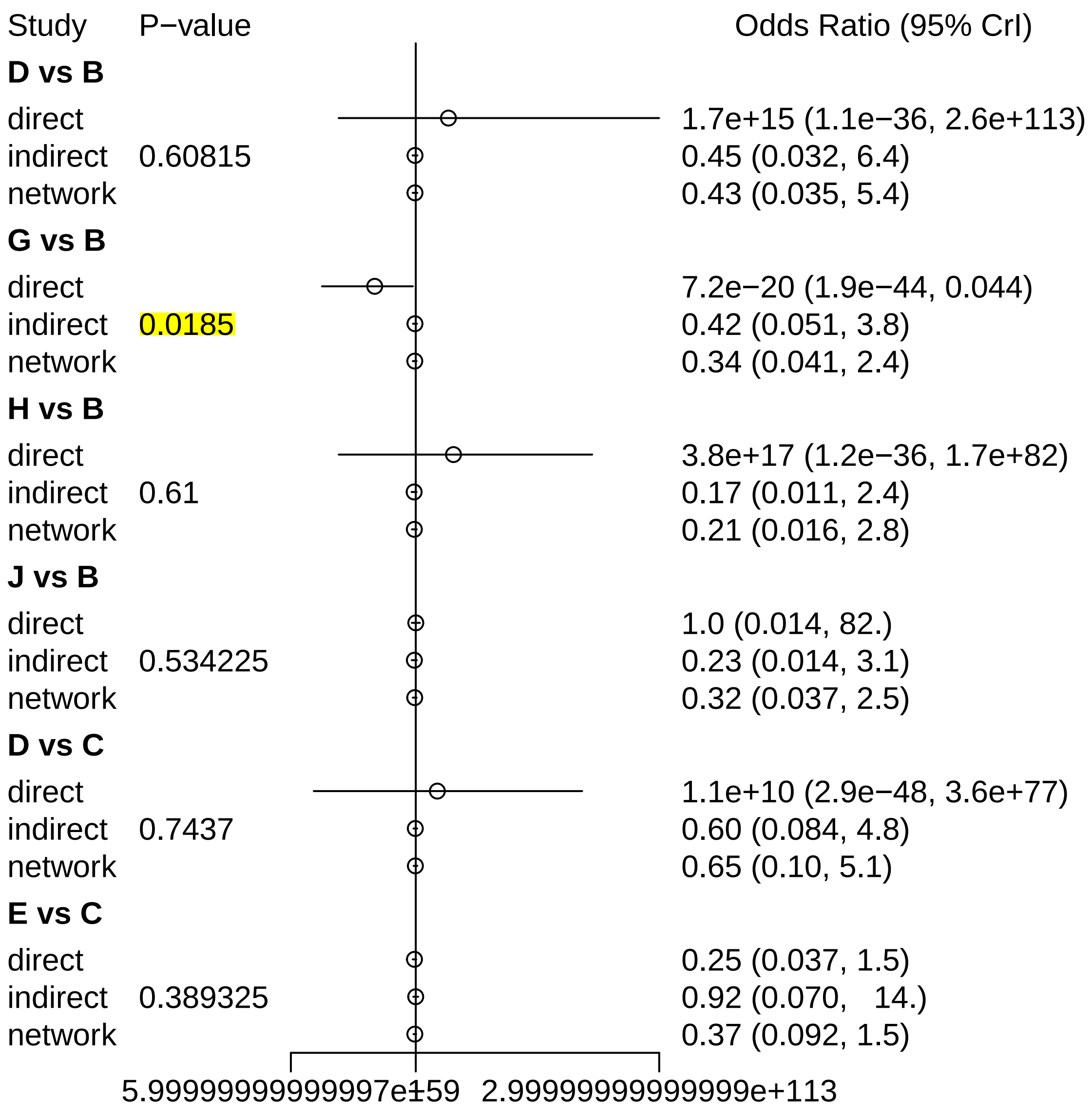
Study
P−value
Odds Ratio (95% CrI)
D vs B
direct
1.7e+15 (1.1e−36, 2.6e+113)
indirect
0.60815
0.45 (0.032, 6.4)
network
0.43 (0.035, 5.4)
G vs B
direct
7.2e−20 (1.9e−44, 0.044)
indirect
0.0185
0.42 (0.051, 3.8)
network
0.34 (0.041, 2.4)
H vs B
direct
3.8e+17 (1.2e−36, 1.7e+82)
indirect
0.61
0.17 (0.011, 2.4)
network
0.21 (0.016, 2.8)
J vs B
direct
1.0 (0.014, 82.)
indirect
0.534225
0.23 (0.014, 3.1)
network
0.32 (0.037, 2.5)
D vs C
direct
1.1e+10 (2.9e−48, 3.6e+77)
indirect
0.7437
0.60 (0.084, 4.8)
network
0.65 (0.10, 5.1)
E vs C
direct
0.25 (0.037, 1.5)
indirect
0.389325
0.92 (0.070, 14.)
network
0.37 (0.092, 1.5)
5.99999999999997e−59
1
2.99999999999999e+113

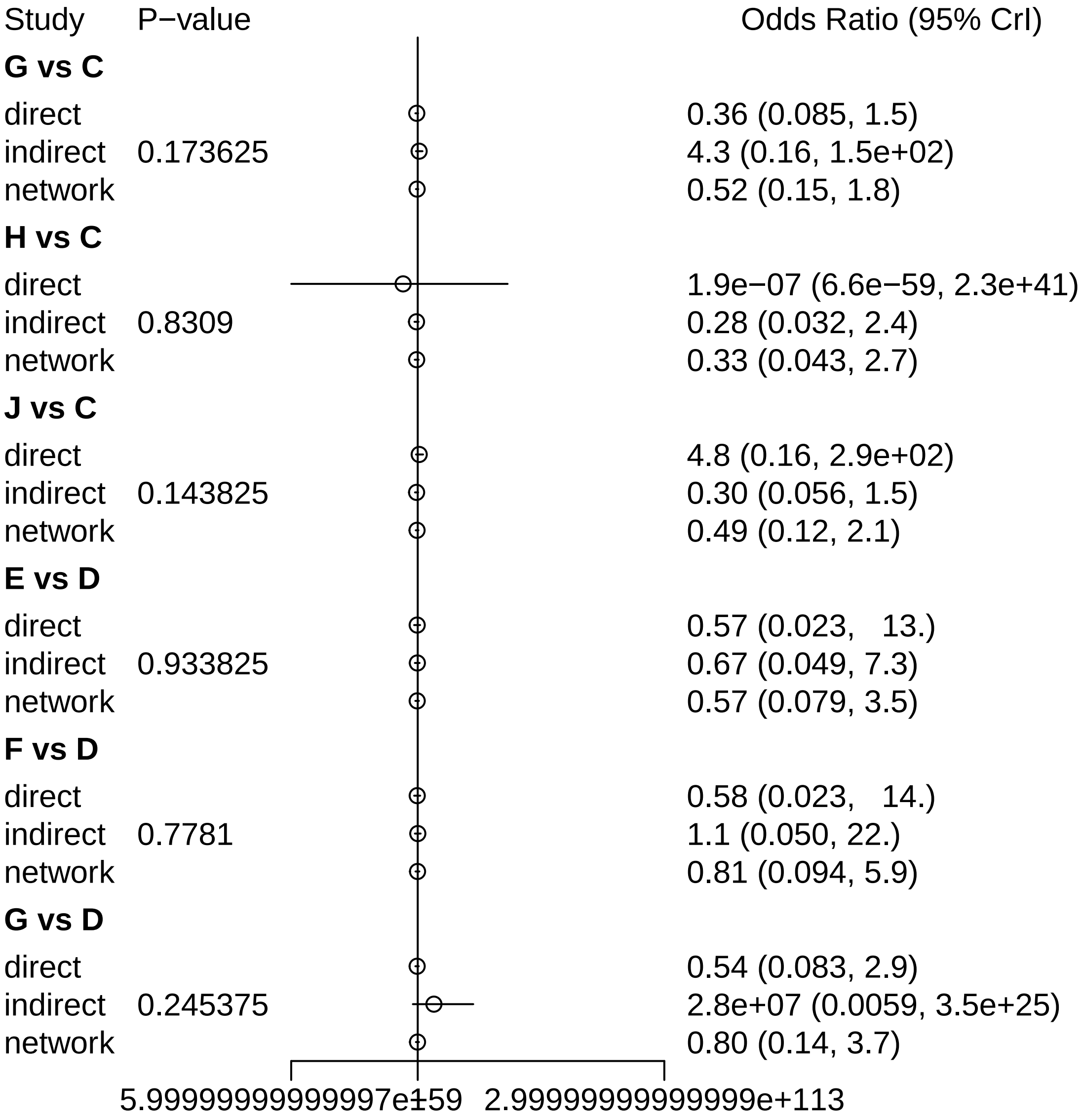
Study P−value Odds Ratio (95% CrI)
G vs C
direct 0.36 (0.085, 1.5)
indirect 0.173625 4.3 (0.16, 1.5e+02)
network 0.52 (0.15, 1.8)
H vs C
direct 1.9e−07 (6.6e−59, 2.3e+41)
indirect 0.8309 0.28 (0.032, 2.4)
network 0.33 (0.043, 2.7)
J vs C
direct 4.8 (0.16, 2.9e+02)
indirect 0.143825 0.30 (0.056, 1.5)
network 0.49 (0.12, 2.1)
E vs D
direct 0.57 (0.023, 13.)
indirect 0.933825 0.67 (0.049, 7.3)
network 0.57 (0.079, 3.5)
F vs D
direct 0.58 (0.023, 14.)
indirect 0.7781 1.1 (0.050, 22.)
network 0.81 (0.094, 5.9)
G vs D
direct 0.54 (0.083, 2.9)
indirect 0.245375 2.8e+07 (0.0059, 3.5e+25)
network 0.80 (0.14, 3.7)
5.99999999999997e−59
1
2.99999999999999e+113

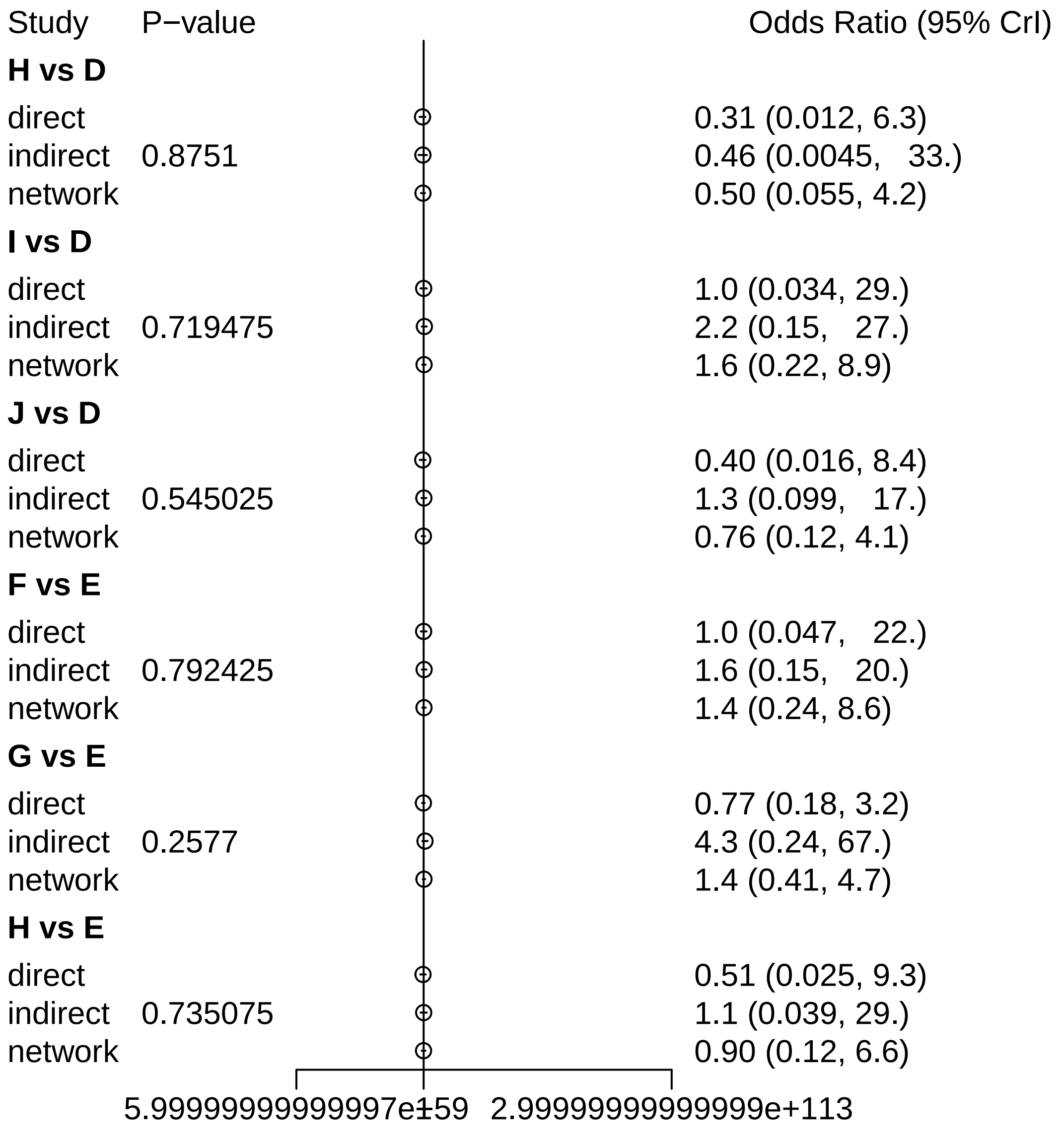
Study
P−value
Odds Ratio (95% CrI)
H vs D
direct
0.31 (0.012, 6.3)
indirect
0.8751
0.46 (0.0045, 33.)
network
0.50 (0.055, 4.2)
I vs D
direct
1.0 (0.034, 29.)
indirect
0.719475
2.2 (0.15, 27.)
network
1.6 (0.22, 8.9)
J vs D
direct
0.40 (0.016, 8.4)
indirect
0.545025
1.3 (0.099, 17.)
network
0.76 (0.12, 4.1)
F vs E
direct
1.0 (0.047, 22.)
indirect
0.792425
1.6 (0.15, 20.)
network
1.4 (0.24, 8.6)
G vs E
direct
0.77 (0.18, 3.2)
indirect
0.2577
4.3 (0.24, 67.)
network
1.4 (0.41, 4.7)
H vs E
direct
0.51 (0.025, 9.3)
indirect
0.735075
1.1 (0.039, 29.)
network
0.90 (0.12, 6.6)
5.99999999999997e−59
1
2.99999999999999e+113

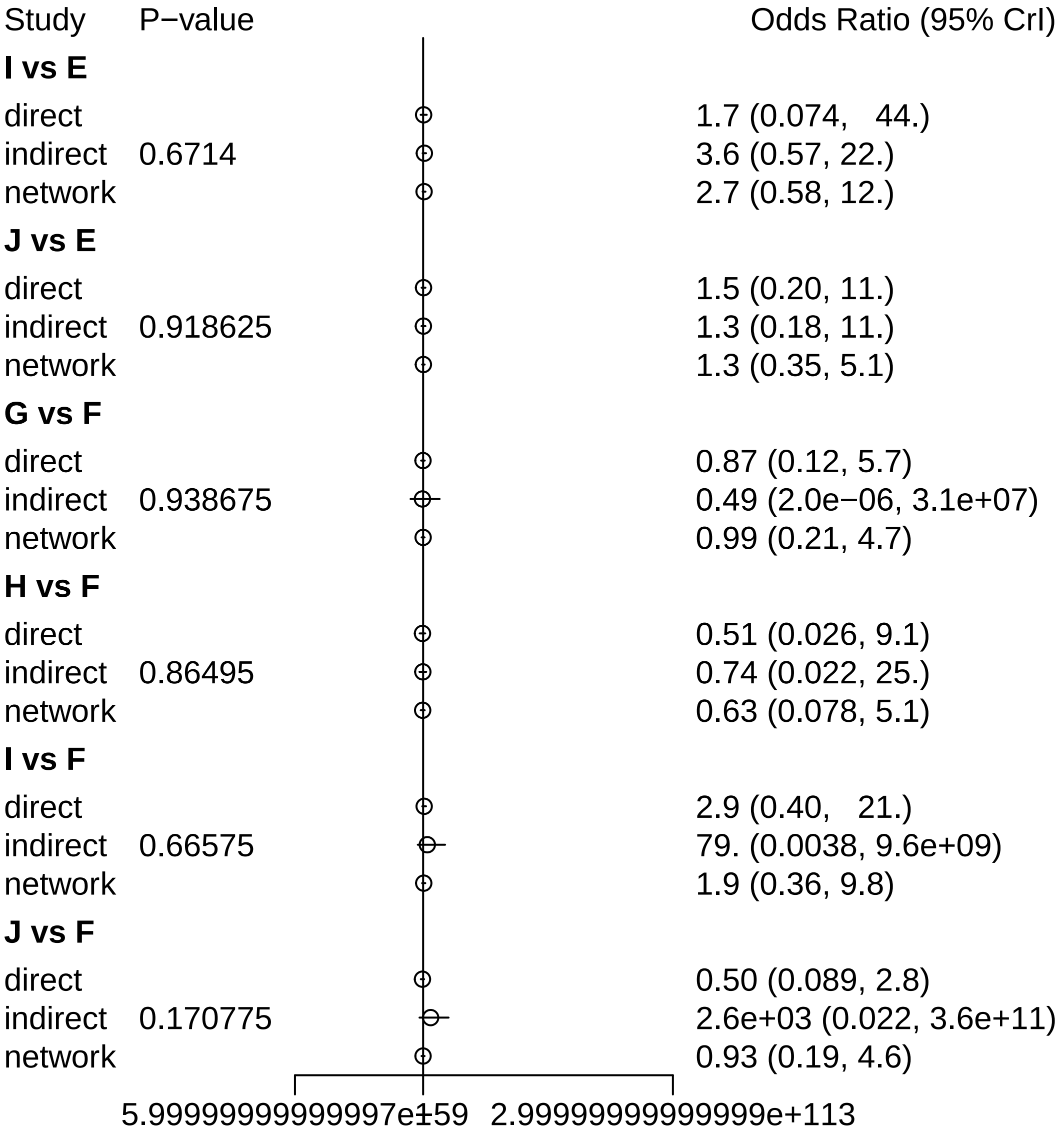
Study
P−value
Odds Ratio (95% CrI)
I vs E
direct
1.7 (0.074, 44.)
indirect
0.6714
3.6 (0.57, 22.)
network
2.7 (0.58, 12.)
J vs E
direct
1.5 (0.20, 11.)
indirect
0.918625
1.3 (0.18, 11.)
network
1.3 (0.35, 5.1)
G vs F
direct
0.87 (0.12, 5.7)
indirect
0.938675
0.49 (2.0e−06, 3.1e+07)
network
0.99 (0.21, 4.7)
H vs F
direct
0.51 (0.026, 9.1)
indirect
0.86495
0.74 (0.022, 25.)
network
0.63 (0.078, 5.1)
I vs F
direct
2.9 (0.40, 21.)
indirect
0.66575
79. (0.0038, 9.6e+09)
network
1.9 (0.36, 9.8)
J vs F
direct
0.50 (0.089, 2.8)
indirect
0.170775
2.6e+03 (0.022, 3.6e+11)
network
0.93 (0.19, 4.6)
5.99999999999997e−59
1
2.99999999999999e+113

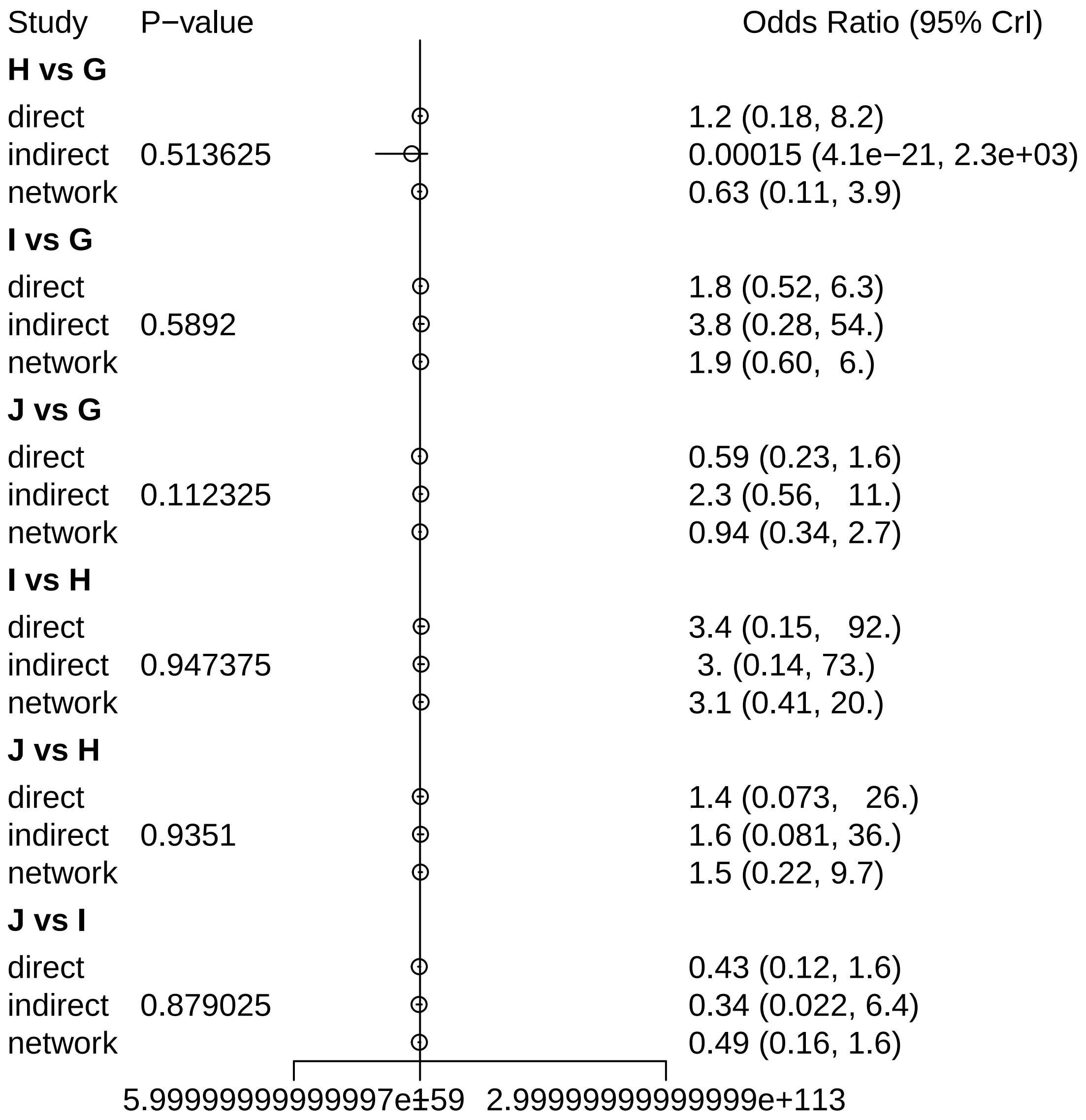
Study
P−value
Odds Ratio (95% CrI)
H vs G
direct
1.2 (0.18, 8.2)
indirect
0.513625
0.00015 (4.1e−21, 2.3e+03)
network
0.63 (0.11, 3.9)
I vs G
direct
1.8 (0.52, 6.3)
indirect
0.5892
3.8 (0.28, 54.)
network
1.9 (0.60, 6.)
J vs G
direct
0.59 (0.23, 1.6)
indirect
0.112325
2.3 (0.56, 11.)
network
0.94 (0.34, 2.7)
I vs H
direct
3.4 (0.15, 92.)
indirect
0.947375
3. (0.14, 73.)
network
3.1 (0.41, 20.)
J vs H
direct
1.4 (0.073, 26.)
indirect
0.9351
1.6 (0.081, 36.)
network
1.5 (0.22, 9.7)
J vs I
direct
0.43 (0.12, 1.6)
indirect
0.879025
0.34 (0.022, 6.4)
network
0.49 (0.16, 1.6)
5.99999999999997e−59
1
2.99999999999999e+113
